# How publics in small-island states view climate change and international responses to it

**DOI:** 10.1073/pnas.2415324122

**Published:** 2025-07-25

**Authors:** Matto Mildenberger, Sara M. Constantino, Paasha Mahdavi, Parrish Bergquist, Gabriel De Roche, Emma Franzblau, Cesar Martinez-Alvarez, Ingmar Sturm

**Affiliations:** ^a^Department of Political Science, University of California, Santa Barbara, CA 93106; ^b^Department of Environmental Social Sciences, Doerr School of Sustainability, Stanford University, Stanford, CA 94305; ^c^Department of Political Science, University of Pennsylvania, Philadelphia, PA 19104; ^d^Department of Political Science, University of California, San Diego, CA 92093; ^e^Department of Political Science, University of California, Los Angeles, CA 90095

**Keywords:** climate change, small-island states, adaptation, public opinion

## Abstract

Climate change caused by carbon pollution from the world’s largest economies poses an existential threat to small-island states and territories this century. These places bear virtually no responsibility for climate change but will face sea-level rise, fresh water resource degradation, and intensified storms that will kill or dislocate exposed publics, and damage local economies. To alleviate this crisis, the global community has begun discussing who is responsible for climate mitigation and adaptation costs for those affected by climate change, in addition to continued debates around the distribution of responsibility for climate change. Missing from this analysis, however, are systematic efforts to elicit the preferences and perceptions of publics in these threatened small-island states and territories. Here, we report results from a large-sample (n = 14,710) cross-national survey of publics living in climate-vulnerable states and territories, conducted in June–July 2022. By quota sampling through Facebook’s ad platform, we generate survey samples at the national or territorial level for publics in 55 small-island states, territories, and subnational regions in the South Pacific, Indian Ocean, and Caribbean. We find widespread awareness and concern about the threat posed by climate change and sea-level rise, in contrast to what existing research finds in the Global North. We also find that climate-vulnerable publics believe their home governments, large polluters, and former colonial powers are all responsible for helping to manage the climate crisis, irrespective of these actors’ relative carbon emissions. These findings fill an important gap by depicting climate beliefs among the communities at the frontlines of climate change.

## Significance

The success of climate adaptation and resilience efforts in vulnerable regions will be shaped by public awareness and acceptance of proposed solutions. Yet for publics in small-island states and territories, who represent some of the world’s most climate-exposed populations, there has been no systematic effort to elicit public preferences and perceptions. We provide a first-of-its-kind survey fielded in 55 small-island states and territories. We find broad acceptance of anthropogenic climate change, but wide variation in perceived climate risks and responsibility over who will resolve climatic threats. In line with an “all-hands-on-deck” approach, publics not only attribute responsibility for solving climate change to major historical and present emitters but also former colonial powers and home-country governments.

Climate change is already causing damages around the world, and addressing these impacts presents enormous political challenges. Many of the most impacted countries or territories have contributed very little to greenhouse gas emissions (1, 2). At the same time, they have limited resources for local adaptation and resilience and, in some cases, may need to consider internal and cross-border relocation of entire communities (3). Climate-induced sea-level rise threatens the sovereign existence of at least five countries by 2100 (Maldives, Tuvalu, the Marshall Islands, Nauru, and Kiribati), and is making large areas uninhabitable in dozens of other countries (IPCC 2022). Effective and equitable adaptation to these climate impacts presents enormous political challenges rooted in state capacity, intergovernmental relations, and individual preferences and behaviors. For instance, adaptation will require massive financial and institutional investments over the coming decades, particularly in developing countries at risk of significant damages from climate change (UNEP 2022). The design and success of these measures will depend on the institutional capacities of climate-vulnerable states, collaboration between governments, and public perceptions and cooperation.

Scholars have paid limited attention to the attitudes and perceptions of the populations most affected by climate change, despite their pivotal role in climate governance. Millions of people around the world have been surveyed about their climate change attitudes and policy preferences over the past two decades (4, 5). A recent comprehensive review of all cross-national climate opinion data (consisting of over 100 surveys of 3.5 million respondents across 164 countries) found that 30 countries and 50 subnational units have never been included in systematic cross-national public opinion samples (6). Remarkably, this set of countries and regions are also the most vulnerable to climate change impacts (7, 8). For instance, nearly all missing countries are small-island states in the Pacific and Indian Oceans and nearly all missing subnational units are either Arctic or desert regions. Moreover, of countries and territories in Oceania, only Australia and New Zealand have ever been included in a cross-national survey (*SI Appendix* includes a summary of these data). In part, this empirical gap is a function of the difficulties associated with surveying remote or small communities, resulting in a lack of data on the climate beliefs and preferences of many of the communities at the frontlines of climate change. Addressing this empirical gap is critical to global efforts to mitigate climate harms because small, vulnerable countries have a potentially outsized influence on international climate policy issues (9). Weak states can play a particularly important role when their salient issues intersect with domestic debates in powerful countries, and when moral and distributional concerns are at stake. When it comes to small-island states and climate change, both conditions are met. Even though public opinion in these countries may not be the dominant factor shaping global climate negotiations, domestic policy preferences are likely to carry outsized weight—both empirically and normatively—making understanding these preferences a priority.

Local engagement with policies to reduce the impacts of environmental hazards depends on a variety of factors (10, 11), two of which are of chief interest here. First, local perceptions of climate harms may play a role in international cooperation on climate policy (12, 13). In the Global North, many people feel a high degree of psychological distance from climate change. They view it as a temporally or spatially distant phenomenon that will largely affect people who are culturally distinct from them (14–17). In turn, psychological distance has been cited as a reason for low support for climate policy (18, 19). In this paper, we examine whether residents of small-island states view climate change as a proximate or a distant phenomenon. Second, distributional concerns affect individual-level perceptions of responsibility for causing and addressing climate change (20–22). Distributional questions are central to global climate policy discussions around who should manage and finance climate change mitigation and adaptation programs. The common but differentiated responsibilities (CBDR) Framework has guided climate negotiations since the formation of the United Nations Framework Convention on Climate Change (23, 24). Under this framework, the largest historical emitters are portrayed as most responsible for addressing climate change, due to their outsize role in causing it. The distribution of responsibility may also follow patterns consistent with existing explanations for preferences over foreign aid. For example, individuals in developing countries tend to view foreign aid by colonial powers as a form of reparations for past damages that they caused (25, 26). Likewise, climate-vulnerable countries might hold their former colonial power responsible for addressing climate harms regardless of their contribution to causing the problem. Here, we explore differences in how residents of the most climate-vulnerable countries view the distribution of responsibility for causing and addressing climate change.

In this paper, we examine how residents of small-island states understand climate change and its associated risks, experience its impacts, and perceive the distribution of responsibility for causing and addressing climate change. To do so, we report results from a large-n cross-national public opinion survey of small-island states and territories, conducted in June–July 2022 (n = 14,710). This survey used Facebook ad-based quotas to generate diverse samples at the national or territorial level for publics in 55 small-island states, territories, and subnational regions in the South Pacific, Indian Ocean, and Caribbean. All respondents completed a multimodule survey instrument that included context-specific questions on experiences with extreme weather and other climate impacts, and experiences and preferences around local, national, and international adaptation policies.

Our results show almost universal acceptance of anthropogenic climate change (ranging from 89 to 100%) as well as very high levels of concern about climate-linked environmental threats—including extreme weather events, sea-level rise, coastal erosion and, to a lesser extent, drinking water contamination. We do not find evidence for a strong gradient of perceived vulnerability, whereby other communities or countries are perceived as more or less vulnerable than respondents themselves. As a secondary analysis, we find little evidence that small-island residents have preferences that are structured along the lines of the CBDR framework, whereby countries are expected to contribute to climate change mitigation and adaptation efforts in proportion to their current and historic greenhouse gas emissions (23). Instead, we find evidence of an “all hands on deck” approach where publics also attribute proportionally high responsibility for solving climate change to former colonial powers and their home-country governments, even relative to these countries’ greenhouse gas contributions. We also find that providing information about relative CO_2_ emissions contributions does little to shift respondents toward a view of proportional responsibility.

## Results

With our survey we sought to understand how the residents of small-island states and territories view and understand climate change and to gauge their preferences regarding how their own governments and the international community should address the changing climate. To this end, we measured respondents’ experience and perceptions of climate risks and attribution of responsibility to different countries for causing and addressing climate change.

### Perceptions of the Future and Climate Salience.

To understand how salient the issue of climate change is to residents of these communities, we first evaluated respondents’ expectations about their futures prior to any mention of climate change, extreme weather, or adaptation. We asked respondents whether they imagined their community being better or worse off in 20 y. We find substantial variation in respondents’ optimism about the future of their communities (*SI Appendix*, Fig. S2), with 58% pessimistic about the future in Aruba to only 12% in St. Kitts and Nevis. This variability extends across the most low-lying countries in the sample. For instance, 53% of Marshall Islanders and 49% of Nauruans believe their community will be worse off in years, whereas a plurality of Maldivians (50%) and a majority of Tuvaluans (59%) and Kiribatians (55%) believe their community will be better off in 20 y.

We asked each respondent to offer a brief explanation of their optimism or pessimism about the future, which allowed us to understand whether respondents consider environment and climate change when assessing the future of their communities.^*^ Factors raised by respondents varied widely, from pessimism about governance to the effects of drug use and weakening economic opportunities. About 11.5% of respondents directly offered climate change, climate policy, sea-level rise, extreme weather, or other environmental considerations (e.g. air pollution, water quality) as one of the most important reasons their community would be better or worse off. This level of concern is larger than surveys in the global North have found; for example, Gallup has consistently found that only 2% of Americans choose “environment and climate change” as the most important problem facing their country today.^†^

### Perceptions of Climate Risk.

**Fig. 1.**
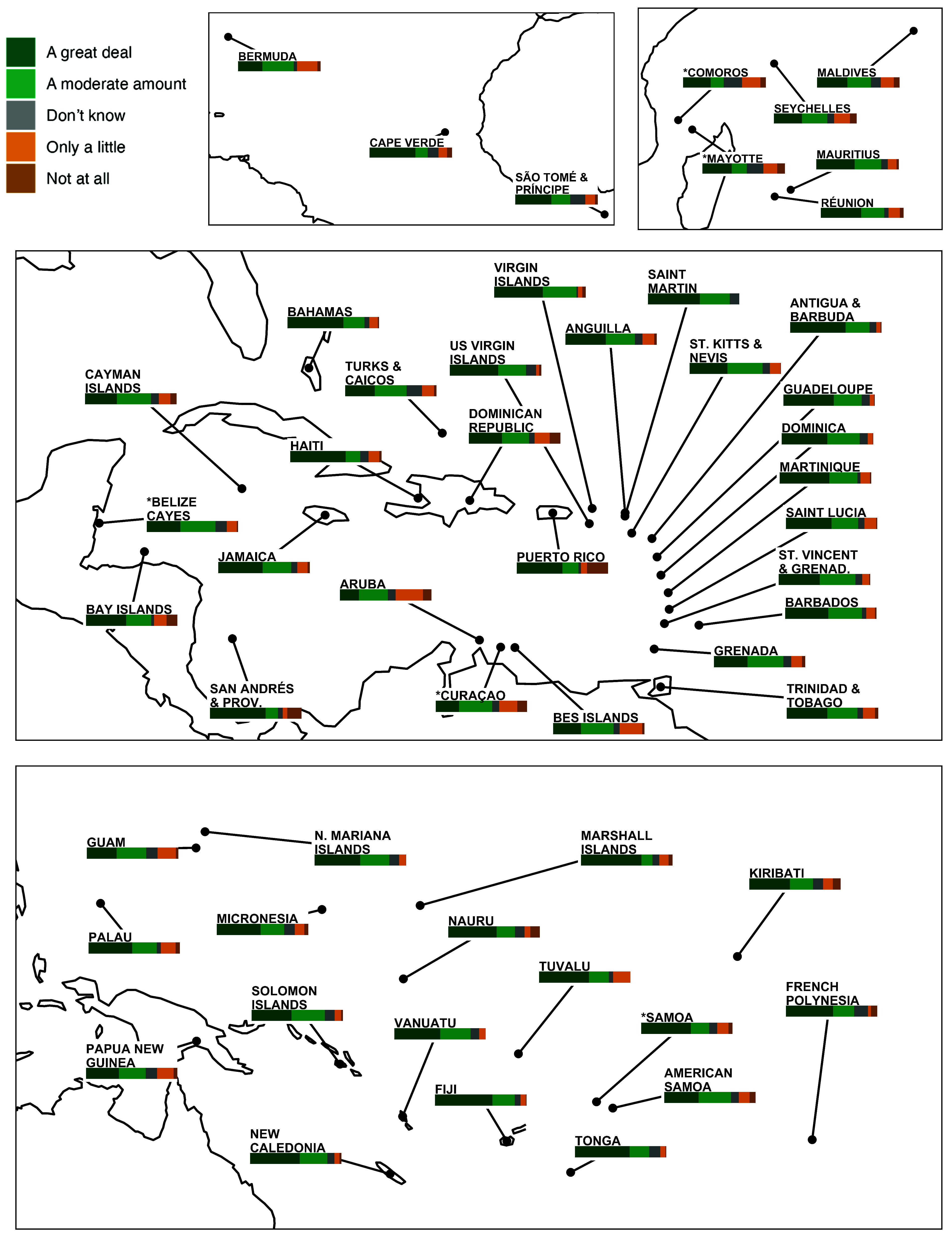
Perceptions of vulnerability to extreme weather. Respondents were asked, “How much do you think extreme weather (like extreme heat, drought, severe storms, floods, hurricanes, or wildfires) will harm people in your community in the next 5 y?” Country/territory-level average responses are shown, with weights to adjust for age and gender imbalances in the samples.

When asked specifically about climate change, respondents expressed a high degree of understanding and alarm about the issue. We found near-universal acceptance of anthropogenic climate change in our samples (*SI Appendix*, Fig. S3), ranging from a low of 89% in Anguilla to 100% in our Marshall Islands and Turks and Caicos samples. By comparison, around the time our study was fielded, only 56% of the US public believed that climate change is caused by human activity (27). Large majorities in every country or territory in our survey are also worried about their personal vulnerability to extreme weather (Fig. 1) as well as sea-level rise and coastal erosion (*SI Appendix*, Fig. S4). In some of the world’s most climate-vulnerable, low-lying islands, these concerns are near universal. In some countries (Nauru, Kiribati, Marshall Islands), majorities also report that their drinking water has already been impacted by sea-level rise, while in many other countries over 25% of the population reports that their drinking water has been contaminated (*SI Appendix*, Fig. S5). In *SI Appendix*, Table S4, we explore the predictors of low concern, finding that being male, not self-identifying as indigenous and perceiving oneself as wealthier are statistically and substantively important correlates of low concern; by contrast, living near to the coast and/or at sea-level are not significantly predictive.

**Fig. 2.**
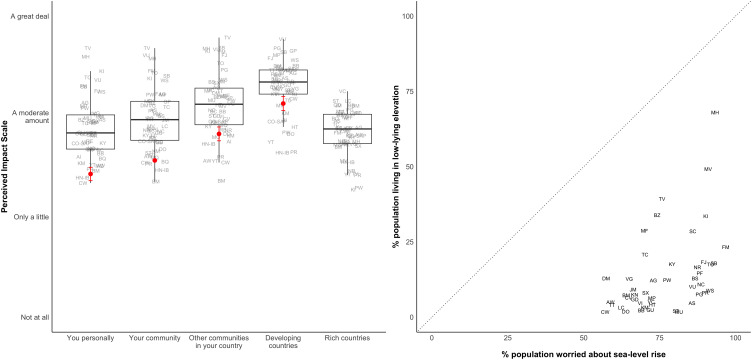
Perceived impact of climate change on select groups and Relationship between objective risk indicators and subjective risk perceptions. *Left*: The figure shows country/territory-level weighted average responses to the following survey question: *How much do you think the following people or groups experienced the impacts of climate change up to this point?* To avoid survey fatigue, respondents were randomly assigned to either receive the sea-level rise or the climate change impact question. Y-axis is a scale from 1 (Not at all) to 4 (A great deal). Random jitter has been applied within each column for visual assistance. Points in red show the average response for the United States. *Right*: The figure shows the fraction of each country or territory’s population living in areas where elevation is below 5 m (from the 2015 World Bank Development Indicators) with the fraction of survey respondents indicating they are very or somewhat worried about sea-level rise or coastal erosion in their local area. The question wording is: *How worried are you about sea-level rise or coastal erosion in your local area?* Country/territory means are weighted.

Broadly, we also find that residents of small-island states feel that climate change is quite proximate, particularly in comparison with publics in wealthy countries such as the United States. We examine psychological distance from climate change by asking whether climate change will impact a series of groups that vary in proximity to survey respondents. In response to this type of question, Americans believe themselves to be relatively insulated from climate change, while they believe more spatially and temporally distant groups to be more impacted (28). To assess whether small-island residents perceive a similar gradient, we modeled our question on a survey item asked in the US context and show responses from a US sample (27) alongside those from our sample. As the figure shows, US respondents view themselves as less exposed than other communities within the United States and developing countries. In contrast, our respondents view most groups to be similarly exposed to climate impacts, with greatest exposure for developing countries (*Left* panel of Fig. 2). For half the sample, we asked the same question about impact, but instead focused on whether sea-level rise (rather than climate change) would impact each group. Here, we find a weaker pattern (*SI Appendix*, Fig. S6). Respondents report, on average, limited past experience with sea-level rise and a stronger gradient of perceived severity comparable to that reported by Americans. This could be because of greater awareness and concern about climate change among residents of small-island states relative to American respondents, and the many impacts encompassed by climate change, e.g., heatwaves, drought, water contamination, or similar. Understanding the multifaceted ways in which climate-vulnerable populations in the global South understand their future risks is thus a critical area for future research. However, our data already emphasize that the populations most vulnerable to climate impacts recognize themselves as such but do not consider the rest of the world to be insulated from climate risks.

How does respondents’ psychological proximity to climate impacts compare with their objective risk exposure? In the Global North, people tend to perceive climate impacts as temporally and spatially distant, even in places where objective risk exposure might suggest otherwise. To assess whether this holds in our sample countries, the *Right* panel of Fig. 2 compares the percentage of the population worried about sea level rise in a given country with an objective proxy for that country’s vulnerability to sea-level rise: the percentage of the population currently living in low-lying areas. All geographic units in our sample are located well below the 45-degree line. This indicates that publics are more worried about sea-level rise in their local area than would be suggested by the fraction of residents living in low-lying areas.^‡^ This finding complements the results shown in the *Left* panel of Fig. 2: Just as the gradient of perceived risk is not particularly strong as the distance from the respondent increases (i.e., moving from “you personally” to “your community” to “other communities in your area”), we also do not find evidence that respondents discern hyperlocalized differences in vulnerability within their own countries.

### Exploring the Distribution of Responsibility for Causing and Addressing Climate Change.

Under the CBDR axiom that has been central to international climate negotiations (23, 24), the largest historical emitters are portrayed as most responsible for mitigation. Public opinion in vulnerable regions that assigns responsibility for addressing climate change to those most responsible for causing it would help explain whether this idea, prominent in climate negotiations, will translate effectively into policy decisions on the ground. If respondents view the largest emitters as most responsible for causing and addressing climate change, their preferences would be in narrow alignment with the CBDR framework.

However, it is also possible that responsibility for climate damages is viewed no differently than international responsibility for any general domestic economic problems, and is structured by a more complex set of distributional concerns. Research on foreign aid, for example, has shown that people in developing countries tend to view aid by colonial powers as a form of reparations for past damages they caused that explain a range of economic maladies in the present (25, 26). If publics in climate-vulnerable countries hold their colonial powers responsible for addressing climate harms, their preferences would be consistent with this broader literature on determinants of foreign aid. Additionally, while most colonial powers are also large historical or current emitters (United States, United Kingdom, Germany), this is not universally true (Spain, Portugal). In general, publics in climate-vulnerable countries may attribute greater responsibility to former colonial powers for causing climate change than would be the case based on emissions calculations alone.

#### Descriptive evidence of responsibility attribution.

**Fig. 3.**
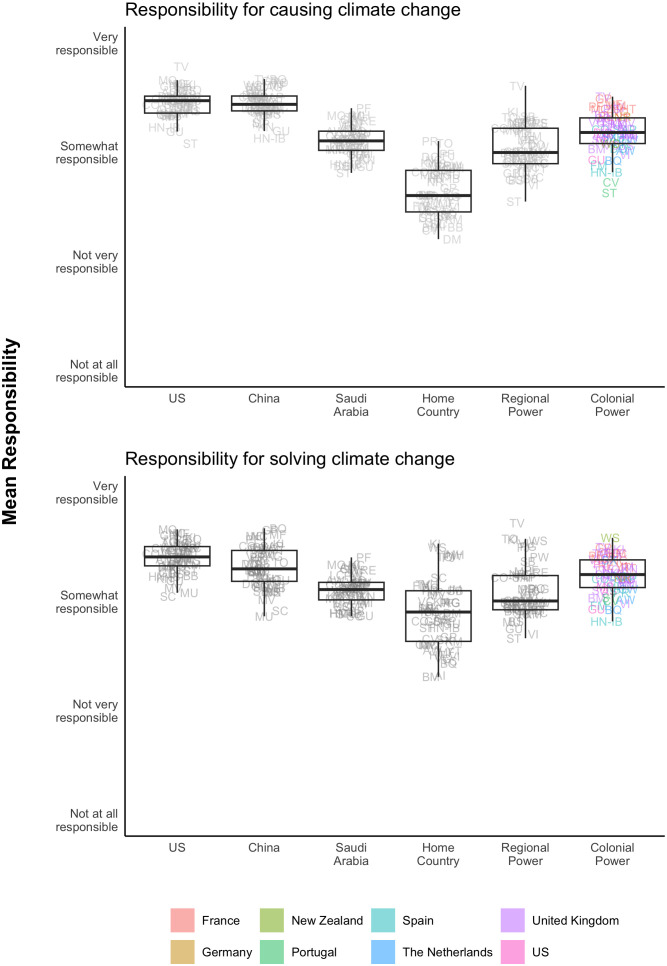
Attribution of responsibility to select countries for causing and for solving climate change. The figure shows responses to the questions, “When you think of climate change and its impacts on your country, how responsible do you think each of the following countries is for causing the problem of climate change?” and “When you think of climate change and its impacts on your country, how responsible do you think each of the following countries is for finding a solution to the problem of climate change?” Points are labeled by country and territory and reflect the mean response in each place, weighted to adjust for imbalances in gender and age distributions in the sample relative to the national population. Random jitter is applied within each column for visual clarity.

We first explore public attributions of responsibility for climate change across a set of countries designed to capture the conceptual distinctions highlighted by the CBDR and reparations frameworks. We asked all respondents to indicate how responsible select countries are for causing and addressing climate change. All respondents were asked to assess US, Chinese, and Saudi Arabian responsibility. These three countries represent high current or historic emitters as a function of domestic economic production, or as a function of global oil production. Respondents were also asked to assess the responsibility of their home country, a specific regional power, and their specific colonial power.^§^Fig. 3 shows attributions of responsibility for causing (*Top* panel) and solving (*Bottom* panel) climate change.

We do not find strong evidence that publics limit their perception of responsibility according to the objective historical emissions of different countries. While respondents view the United States and China as more responsible for causing the climate crisis than their colonial power, as shown in the *Top* panel of Fig. 3, these differences are much smaller than what might be expected based on historical emissions (mean values: $\mu_{\text{US}}=3.45$, $\mu_{\text{China}}=3.45$, and $\mu_{\text{colonial powers}}=3.18$). We also find that respondents in climate-vulnerable countries hold their colonial powers almost as responsible as the United States for solving the climate crisis ($\mu_{\text{colonial powers}}$ = 3.2, $\mu_{\text{US}}$ and $\mu_{\text{China}}=3.3$), as shown in the *Bottom* panel of Fig. 3. This relative parity exists despite dramatic imbalances in emissions contributions between the United States and the colonial powers included in the survey. For example, the United States has contributed more than five times as much carbon pollution into the global atmosphere as the United Kingdom since the beginning of the industrial revolution. Yet, we find that publics in 17 of the 26 former British colonies view the United Kingdom as just as, if not more, responsible for solving climate change as the United States. Data constraints limit our ability to disentangle whether these results are, for example, supportive of the idea that people in former colonies may view climate action as a form of reparation for past damage, or whether attributing blame/responsibility to former colonial powers is a kind of availability heuristic that captures the role of industrialized countries more broadly in causing climate change. Better understanding these dynamics is a potentially fruitful avenue for future research. Notably, respondents also see their home-country governments as bearing significant responsibility ($\mu_{\text{home country}}=2.9$ on a 4-point Likert scale, where 3 = “somewhat responsible,”), rather than assigning all responsibility to the highest-emitting countries (Fig. 3).^¶^

#### Experimental evidence on responsibility attribution.

To complement these descriptive results, we used an experiment to directly examine the relationship between information about national contributions to greenhouse gas emissions and perceived responsibility for financing climate solutions. We randomly assigned respondents to one of four vignette conditions: 1) information about a country’s historical emissions contributions, 2) information about a country’s current emissions contributions, 3) information about a country’s historical and current emissions contributions, or 4) no information (i.e., our control condition). We also randomized the countries featured in the vignette, selecting countries that cover both high and low cumulative and current emissions (i.e., United States, China, India, Bangladesh, Switzerland, and United Kingdom), in order to examine whether this information impacts attributions of responsibility.^#^ We then asked respondents how much that country should contribute to the global climate aid fund for vulnerable countries. This experiment thus provides a direct test of whether public preferences align with the CBDR framework. Under the CBDR framework, we would expect information about cumulative or historical emissions to increase expected contributions from high-emitting countries (the United States, China, and India), and reduce expected contributions from low-emitting countries (Bangladesh, Switzerland, and the United Kingdom).

**Fig. 4.**
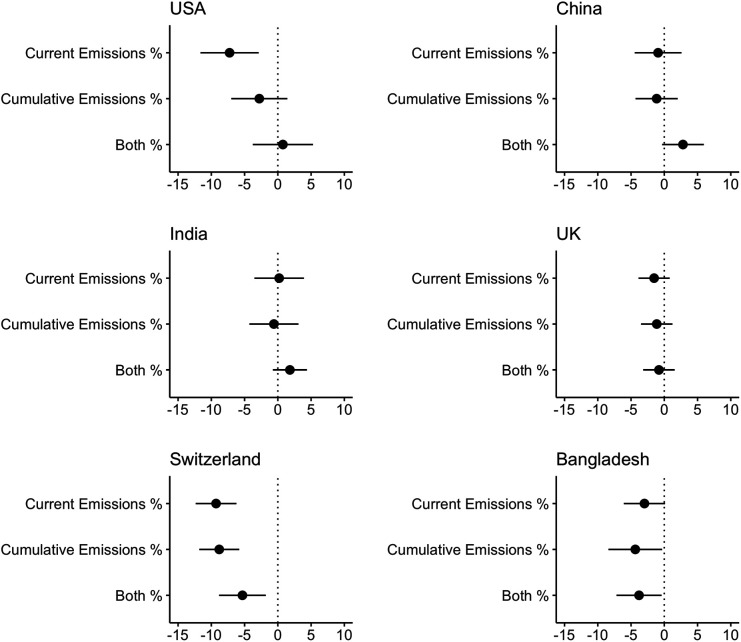
Perceived need for climate finance contributions by specific countries. Each pane shows the effect of exposure to information about current or cumulative carbon pollution contributions on desired contributions from a given country (United States, China, India, United Kingdom, Switzerland, Bangladesh) to the global finance fund. Coefficients are based on country/territory-pooled weighted regression models with SE clustered by country/territory.

Fig. 4 shows the treatment effect of each information condition on the desired contribution from each country in our experiment. Our results are surprising. As expected, revealing the relatively low emissions contributions of both Switzerland and Bangladesh reduces the desired contributions from both countries. However, we do not see evidence that information about countries with higher cumulative emissions contributions to the climate crisis or higher current contributions increases the perceived obligation of those countries to contribute more to climate adaptation finance. We suspect this may be because individuals in our sample attribute to the highest emitters strong responsibility for causing climate change and for contributing to solutions, such that information about their high contributions does not spur an increase in desired climate finance contributions. Indeed, expected contributions to the adaptation fund from the United States, China, and (to a slightly lesser extent) the United Kingdom were higher, even within the group that received no information about emissions (*SI Appendix*, Fig. S9). On the other hand, against this prior perception, revealing the US current emissions contributions decreases desired contributions from the United States slightly, perhaps against an inflated perception of the fraction of global carbon pollution currently being released by the United States. Thus, explicitly mentioning emissions contributions appears to reduce expected contributions to climate finance by low-emitting countries (reflected in the negative coefficient estimates in Fig. 4), but we do not find evidence that it raises expected contributions from high-emitting countries.

## Conclusion

Communities around the world are already experiencing the impacts of climate change, including sea-level rise and extreme weather events, among others. Given the central role that local governments and communities play to foster effective climate adaptation (30, 31), we should expect that public opinion support is a key factor facilitating the implementation of such policies among the most vulnerable countries. Nonetheless, publics in small-island states who are on the frontlines of climate change have been mostly absent from studies that gauge the preferences and attitudes of individuals toward climate change vulnerability, mitigation, and adaptation. As a result, we know little about their perceptions of their own climate vulnerability and their attitudes toward international and domestic adaptation policy.

This paper aims to address these gaps through a cross-national public opinion survey of small-island states and territories, which employed Facebook ad-based quotas to obtain diverse samples in 55 states and territories. We focus on perceptions of vulnerability to climate change impacts and the distribution of responsibility for causing and addressing climate change.

We find that respondents in small-island states and territories have an almost universal acceptance of anthropogenic climate change as well as high levels of concern about the environmental impacts associated with it. They view these impacts as quite proximate, not only for themselves but also for distant communities and more developed countries. This contrasts with American respondents, who report lower personal risk perceptions and a stronger gradient of perceived proximity to climate risks between themselves and other communities. This relative parity in perceived vulnerability to climate change is somewhat mirrored in our respondents’ attribution of responsibility for causing and addressing climate change. In a secondary analysis, we find that low-lying island residents attribute approximately equal responsibility for causing and addressing climate change to the highest emitting countries and to their colonial powers, despite stark differences in their respective contributions to climate change. Interestingly, this attribution of responsibility for causing climate change does not translate to a view that their own countries should not contribute to climate solutions. Instead, low-lying island residents perceive their own countries as somewhat responsible for addressing climate change, and only slightly less responsible than the highest emitters and colonial powers. Moreover, explicitly providing information about current and historical greenhouse gas emissions does not cause an increase in expected contributions from the highest emitting countries.

We do not interpret these findings to imply that the “CBDR” framework should be abandoned as a principle. It is still a hallmark of equity and fairness that is critical for constructive and inclusive international environmental negotiations (32, 33). Rather, these findings demonstrate that publics in climate-vulnerable regions prefer a more “all hands on deck” approach given the urgency of the climate crisis in small-island states and territories. Leaders from these states would do well to respond to their constituents’ preferences, by pushing for more ambitious policy on climate adaptation and resilience by large current polluters, former colonial powers, and by their own governments.

## Materials and Methods

### Survey Data in the Global South.

Our previous work has comprehensively assessed the availability of survey data from the most climate-vulnerable areas of the world (6). That work involved generating a master database of all publicly released cross-national opinion surveys that include one or more climate-related questions between 2000 and the present. Overall, we found 101 surveys that asked the same question in two or more countries, spanning 164 countries overall (described in detail in *SI Appendix*). However, climate-vulnerable countries (and territories) are systematically absent from this dataset. Of the small-island states and territories surveyed, only a handful have ever been included in previous research. Notably, the Latin American Public Opinion Project (LAPOP) survey in 2016 included some smaller Caribbean island nations. However, few South Pacific countries have ever been included in cross-national surveys, including relatively populous countries like Fiji, nor have other countries vulnerable to sea-level rise, including the Maldives in the Indian Ocean. Among territories and dependencies, there is even less coverage. A few European surveys occasionally include a handful of respondents from French or Dutch dependencies in the Caribbean and Indian Ocean, but never a structured sample that allows for these subnational units to be analyzed independently. The SI summarizes countries that have never been included in a climate survey. This results in a paradoxical outcome: the very places that are most at risk of climate change are also the places that have been least considered.

We used Facebook advertising to quota sample the public in every small-island state and territory worldwide. Facebook advertising provides new opportunities for researchers to cheaply engage diverse survey respondents who are otherwise challenging to recruit into social science research (34, 35). We first describe our sampling frame, including our strategy for setting geographic and demographic survey quotas. We then describe our sampling process, including our approach to quota sampling with Facebook advertisements and our strategy for constructing survey weights. Finally, we outline the structure of our survey instrument.

### Sampling Frame.

We established a separate sampling frame for every small-island state in the world and every small-island territory or dependency (population > 10,000).^‖^*SI Appendix*, Table S2 summarizes these sampled countries and/or territories. For countries or territories with an adult population ≲ 100,000, we generated sampling quotas using joint distributions of age and sex from data available through the United States Census Bureau’s International Database (IDB).^**^ The IDB provides single-year age estimates for males and females in all of the world’s countries, as well as for many of the territories in our sample using National Census Bureau information, surveys, and vital statistics registries, and generates these estimates while accounting for significant events that may affect population totals and distributions. For these countries and territories (adult populations less than 100,000), we generated quotas using the six joint distributions of i) women and ii) men in each of the following age groups: 18 to 29; 30 to 49; 50 and older

For countries and territories with adult populations ≳ 100,000, we obtained the most recent census data from national census bureaus/statistical agencies. On a country-by-country basis, we examined the population distribution across administrative divisions to split the country or territory into geographic quotas, with the goal of sampling individuals residing in both the most densely populated and less densely populated regions of a country or territory according to their relative distribution. In most cases, this meant creating a geographic quota for the administrative division containing the capital city and its environs, and quotas for other administrative divisions that varied in their population density. Within each geographic quota, we analyzed the most recent census data to determine the joint distribution of i) women and ii) men, each in the 18 to 29, 30 to 49, and ≥50 age groups in a given geography. *SI Appendix*, Table S5 summarizes the final geographic quotas for each sampled country or territory.

For every geography, we set the total sampling target at a value of 0.25% of the eligible population (1 in 400), with a floor of 150 (for places where this number was smaller) and a ceiling of 750 (for places where this number was larger). The 750 ceiling was chosen to allow for five survey experimental arms of n = 150 in these larger countries. We then generated quota-specific sample size targets by multiplying the geography’s total sample target by the quota’s probability under the geography’s joint distribution. We provide an example of these targets in *SI Appendix*.

### Sampling Process.

**Fig. 5.**
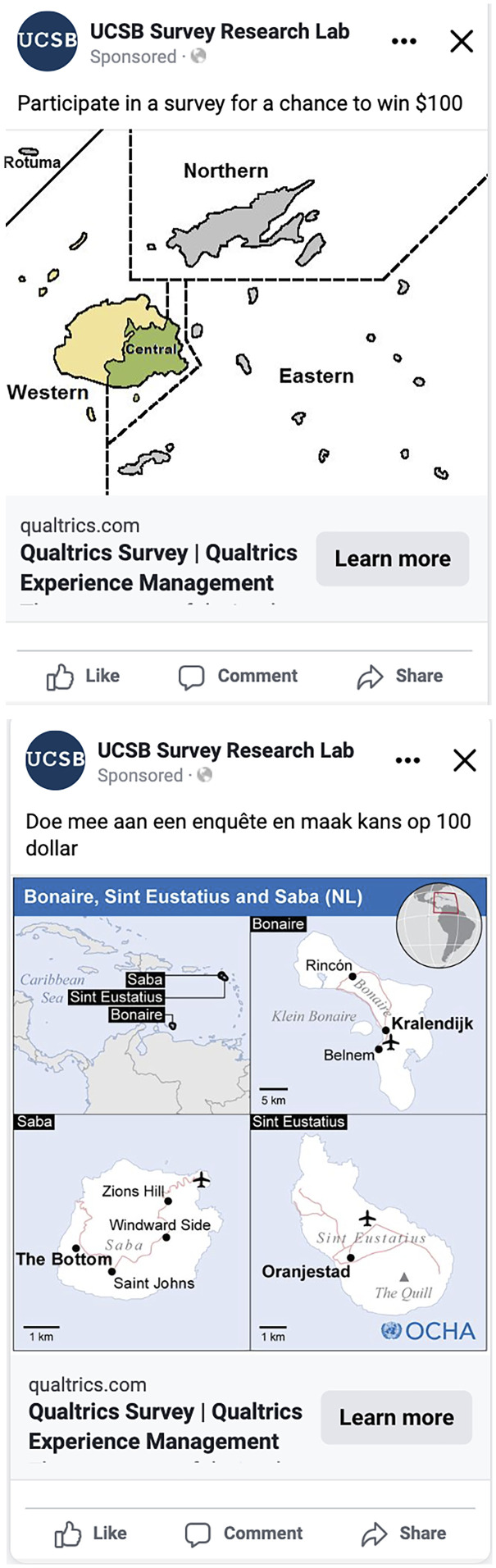
Example Facebook recruitment ads.

We created a Facebook advertising “campaign” for each country or territory in our sample, and then created individual “ad sets” for every quota defined for that geography. Facebook allows users to target specific gender, ages, and regions for advertisements. Two examples of recruitment advertising are shown in Fig. 5. Ads were delivered in one of five languages, depending on the most common spoken language in each territory or country (English, French, Spanish, Portuguese, Dutch). Clicking on the link embedded in the ad directed individuals to our survey splash and consent page, programmed with the Qualtrics survey design tool. Ongoing and parallel work by our research team has worked to validate the use of quota-sampling with Facebook advertisements in the global South (35).

We used the same ad copy for every geography, but varied the recruitment image to be a map of that island state or territory. The advertisements did not specify the topic of the survey. Initially, respondents were invited to participate for a chance to win $100 USD. We explained that we would provide a prize for every 500 individuals who completed the survey, generating a minimum of 1 in 500 odds of winning. At later stages of the recruitment, we increased the prize for underperforming geographies and/or quotas to a 1 in 1,000 chance of winning $500 USD. The link embedded in each ad set included metadata that let us capture information about the Facebook targeting and ad conditions that sent our respondents into the survey, including the value of the prize. For instance, this metadata tells us that an incoming respondent clicked on a Facebook advertisement that had been served to men over the age of 50 in Western Fiji. This metadata also allowed us to tailor each respondent’s survey dynamically to include geography-specific questions or fields.^††^

We sampled in June and July 2022 (specific date ranges varied by country). In *SI Appendix*, Table S6, we summarize our sampling efforts in each country. This includes our total spend on Facebook ads, the number of people potentially served our ads (“Reach”), the number of times an individual was served this ad (“Impressions”) which accounts for individuals that were served the ad multiple times, the number of times the ad link was clicked directing respondents to Qualtrics (“Link Clicks”) and the number of people who completely finished our survey and were redirected back to Facebook (“Conversions”). Conversions are tracked using a Facebook “pixel” embedded in the final survey page, notifying Facebook that the ad-targeted individual completed the desired recruitment task. Note that, while Facebook tracks this task completion, it does not share the identity of individuals with advertisers.

Survey completions declined dramatically over time as we switched resources toward more difficult to sample quotas. We manually turned country-level campaigns on and off to vary the time of day and day of week in which recruitment advertisements were delivered. Initially, we turned off any ad sets for quotas where we hit our benchmark target number of responses for each quota (*Sampling Frame*). An individual contributed to the target response number if they completed at least one question in the final substantive survey block (Block 5 on global climate politics; see *Survey Instrument*). We assessed individuals based on their self-reported age, gender, and quota region where available, or otherwise imputed this information from the Facebook ad that brought them into the survey if they did not complete all demographic questions.^‡‡^

As our resources become more scarce, we established a series of additional conditions under which ad sets were paused. These were 1) turning off ads for quotas that had filled to a minimum level (one quarter of the sample target), as long as this was larger than five respondents; and 2) turning off ads for small geographies where our sample already exceeded 0.25% of the total eligible population (1 in 400 people), and where this 0.25% size was less than the 150 minimum sampling frame quota size. As an example, recruiting 150 respondents from Nauru would require us to sample 1 out of every 44 adult residents of Nauru. Facebook penetration in Nauru is reported at 42.5%, though skewing toward younger residents. This suggests that a target of n = 150 might have required recruiting around 1 of every 20 Nauruan adult Facebook users, an implausibly high sampling rate with this method.

### Sample Sizes + Survey Weights.

Full details of sample size across quotas and levels of survey completion are provided for each country and territory in *SI Appendix*. As a result of response-rate attrition across the survey, the number of complete responses varies by question. We provide the maximum sample size of respondents for questions in each of the survey’s five blocks or modules (see Survey Instrument). Relative to 25,095 respondents who completed the first module, we saw cumulative attrition of 4.3% by the second of five modules, 9.6% by the third module, 13.4% by the fourth of five modules, and 22.1% who completed all survey questions including detailed demographics. Based on these response rates, we constructed weights for every survey respondent to account for demographic imbalances that remained in our sample. While 19,375 individuals answered at least one question in the final block, sufficient demographic information was only answered by 14,710 individuals for weighting. This is the n of the weighted dataset, which we use for all analysis unless otherwise specified. We use iterative proportional fitting, also known as raking, to reweight our sample to match the marginal distributions of age, gender, and geography, using the same joint distributions used to construct our sampling frame. We implement this raking using the svyweight package in R. Countries or territories with missing data from 1 or more quotas (Territory of Curacao, the cayes of Belize, Comoros, Western Samoa, and the Territory of Mayotte) were left unweighted. Countries or territories with less than 50 total complete responses (Tuvalu, Nauru, Anguilla) are analyzed with weights but the small sample sizes here suggest uncertainty around even the weighted sample’s relationship to true population means. For these two sets of countries, we frequently visualize their country means or country codes in blue throughout, to highlight decreased confidence in the plausibly representative nature of the country or territory-level estimates.

### Survey Instrument.

Our survey instrument is summarized in *SI Appendix* and was delivered to respondents using the Qualtrics survey platform. The survey was translated into four additional languages (French, Spanish, Portuguese, Dutch), and delivered to respondents in the language of their Facebook ad. The survey was divided loosely into five module blocks. Block 1 addressed individuals’ perceptions of future well-being and topics of migration generally. This block did not specifically discuss either climate change or environmental stressors. Block 2 addressed experiences with extreme weather broadly. Block 3 addressed climate change attitudes, including questions of attributing extreme weather experiences to climate variables. Block 4 asked questions about experiences with climate adaptation policies, and perceptions of the actors responsible for managing these policies. Block 5 asked questions about global climate politics, including questions related to responsibility for financing climate adaptation projects. This section also queried respondents on the types of financial or legal supports that would help them navigate future environmental changes. Block 6 asked questions about displacement and migration, including questions about attitudes toward receiving migrants from other countries and relocating to other countries. A final question bank asked demographic questions about respondents, followed by an option to enter contact information for the prize drawing.

In Block 5, we also asked a *finance responsibility* survey experiment, where respondents had to indicate how much different countries should be providing into a global climate finance fund. Respondents read a brief paragraph describing this $100 billion USD fund, and then were asked to choose an amount for the referent country to contribute to this fund. The contributing country was randomly assigned (United States, China, India, United Kingdom, Switzerland, Bangladesh). The contribution of that country to carbon pollution was also randomized into one of four conditions. A control condition provided no information about carbon pollution contributions. A second condition gave the fraction of contemporaneous pollution the country is responsible for. A third condition gave the fraction of cumulative pollution the country has been responsible for. A final condition provides both contemporaneous and cumulative statistics. The identity of the contributing country was also randomly assigned across respondents.

Table 1 gives the contemporaneous and cumulative statistics provided in the experiment for each contributing country. Countries were chosen to provide a diversity of present vs. historical emissions contributions. The United States and China have high historic and current carbon pollution levels. India has higher current levels than its cumulative contributions. The United Kingdom has higher cumulative levels than its current contributions. Switzerland and Bangladesh have very low levels on both metrics, but represent both a wealthy and climate-vulnerable country example of this low–low condition.

**Table 1.** Treatment conditions for finance experiment

**Country**	**Now %**	**Cumulative %**
United States	13.54%	24.65%
China	30.65%	13.89%
India	7.02%	3.21%
United Kingdom	0.95%	4.61%
Switzerland	Much less than 1% (0.09%)	Much less than 1% (0.18%)
Bangladesh	Much less than 1% (0.27%)	Much less than 1% (0.09%)

Table provides the contemporaneous % of cumulative % of carbon pollution that a given country is responsible for.

Our survey and research design was approved by the University of California Santa Barbara Office of Research as Protocol #34-23-0660. All respondents provided consent to participate in the study as part of the survey’s splash page, which also provided transparent information on the project.

## Supplementary Material

Appendix 01 (PDF)

## Data Availability

Data and replication materials are available on Harvard Dataverse at https://doi.org/10.7910/DVN/YC97YF (36).

## Footnotes

^*^Our question prompt was: using just a few words: when you imagine your community in 20 y, what is the most important reason you think your community will be better/worse off? This analysis uses a larger sample size of 19,312 respondents who answered the open-ended question, at the very beginning of the survey; many of these individuals later attrited from the survey before providing the demographic information needed for weighting. Restricting analysis to the 14,710 respondents used in the papers’ other analyses, we find a similar 9.6% fraction who mentioned climate change or environment.

^†^https://news.gallup.com/poll/1675/most-important-problem.aspx.

^‡^Of course, respondents may interpret “local area” in a variety of ways and our objective measure of exposure is crude, and does not account for measures taken to protect low-lying communities from sea-level rise or coastal erosion. Both of these factors may explain, to some extent, the pattern we see. The crucial finding is that these climate-vulnerable populations do not underestimate risk, unlike the results observed in the global North (29).

^§^Respondents saw individual country names, not these categories; but the country names provided to respondents varied with their country of residence.

^¶^We formally test differences in average attribution of responsibility with regression models whose results are shown in *SI Appendix*, Table S3.

^#^We also incorporated variation on national wealth and colonial history to ensure that these factors are not driving any effects we see.

^‖^We also included four subnational islands or island archipelagos in our sample: the department of San Andrés and Providencia in Colombia, the department of Bay Islands in Honduras, the US State of Hawaii, and the populated cayes in Belize.

^**^https://www.census.gov/programs-surveys/international-programs/data/tools/international-data-base.html (Accessed 19 July 2022).

^††^Initial ad sets for Anguilla, Bermuda (UK), and the Bahamas had an error in their URL which prevented complete metadata capture (but did not impact the survey as experienced by respondents). This resulted in some partial responses with unknown demographics that could not be imputed. These are detailed in *SI Appendix*.

^‡‡^At this point in our sampling process, we did not assess the geography where a respondent self-reported living, if different from the geography of the Facebook ad that brought them into the survey. However, we account for these differences in our analysis and sample size calculations below. Also, residents of Belizean cayes were inadvertently not shown a list of countries that included Belize, so we must assume their self-reported country based on the FB ad that brought them into the survey.
