# Supplementary material for: How publics in small-island states view climate change and international responses to it
Source: Proc Natl Acad Sci U S A. 2025 Jul 25;122(30):e2415324122. doi: 10.1073/pnas.2415324122 (PMC12318213; doi:10.1073/pnas.2415324122)
Supplement: Supplementary file 1 — Appendix 01 (PDF) [file pnas.2415324122.sapp.pdf]

Supplementary Information for "How publics in small-island states view climate change and international responses to it"

Matto Mildenberger[1], Sara M. Constantino[2], Paasha Mahdavi[1], Parrish Bergquist[3], Gabriel De Roche[4], Emma Franzblau[1], Cesar Martinez-Alvarez[1], and Ingmar Sturm[1]

[1]Department of Political Science, UC Santa Barbara

[2]Doerr School of Sustainability, Stanford University

[3]Department of Political Science, UC San Diego

[4]Department of Political Science, University of Pennsylvania

June 21, 2025

1 Geographic coverage of pre-existing climate survey data

Supplementary Figure 1: **Geographic coverage of publicly-available cross-national public opinion datasets that include at least one climate-related question.** Source data comes from Bergquist et al. (2025), and includes analysis of 101 surveys that ask publics in 164 different countries about climate issues. The figure shows the coverage of our survey data in each country (arrayed along the Y axis) and each year (along the X axis). Dots are shaded to correspond to the number of unique questions available in each country-year. Darker dots represent country-years with relatively thick data.

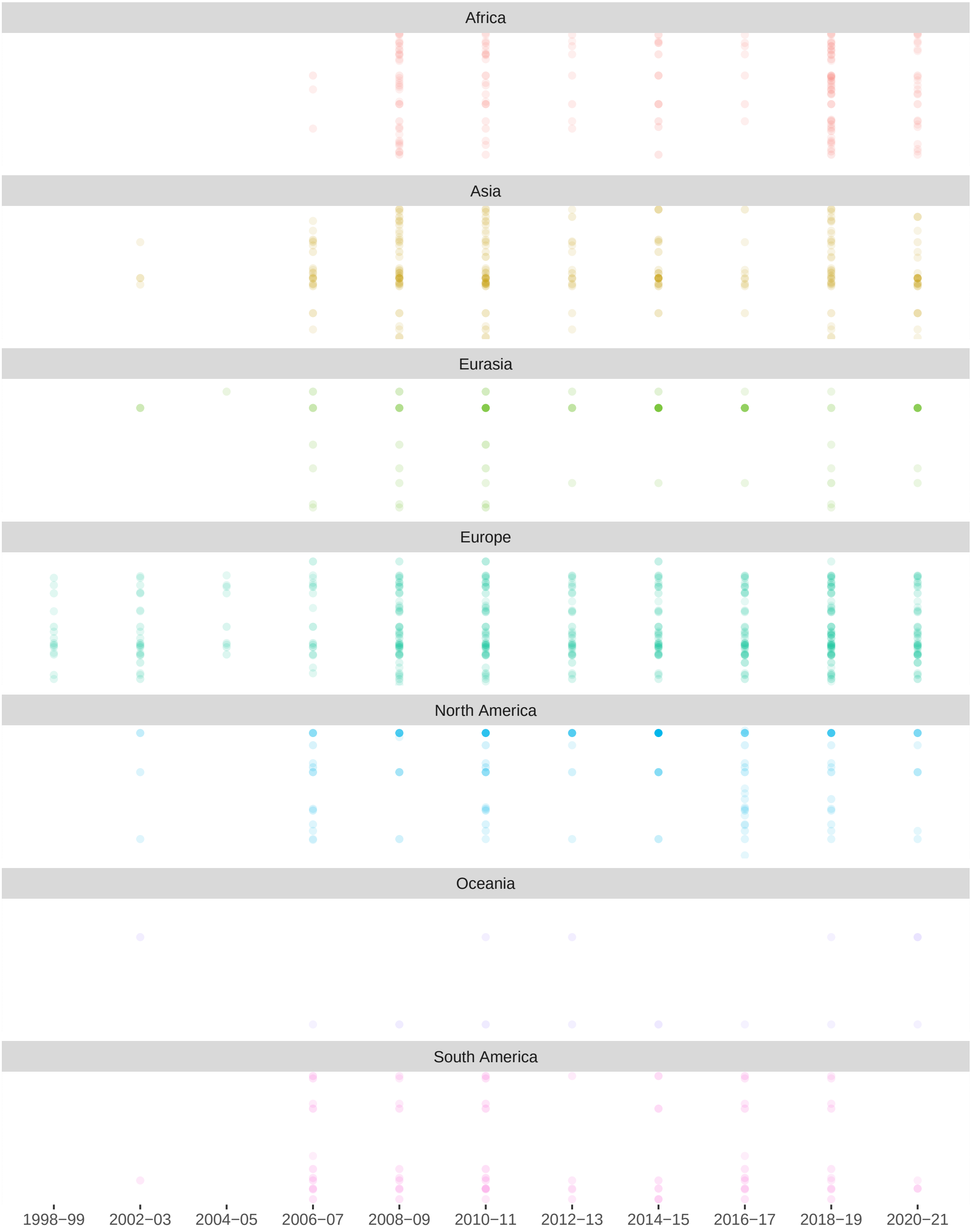

2 Missing Countries from Known Climate Surveys

Angola	Nauru
Bahamas	Oman
Barbados	Palau
Bhutan	Papua New Guinea
Brunei	Samoa
Cook Islands	San Marino
Cuba	Sao Tome and Principe
Equatorial Guinea	Seychelles
Eritrea	Solomon Islands
Fiji	South Sudan
Guinea-Bissau	Suriname
Kiribati	Timor-Leste
North Korea	Tonga
Liechtenstein	Tuvalu
Maldives	Vanuatu
Marshall Islands	Vatican City
Monaco	

Supplementary Table 1: **Countries that have never been included in a cross-national survey that includes at least one question about climate change**

3 Countries and Territories Included in Sampling Frame

Supplementary Table 2: **Small-island states and territories included in sampling frame**. Also provided are the two-character ISO3166 or ISO3166-2 country or territory codes used as country shorthand in figures and tables throughout the text.

Code	Country or Territory	Code	Country or Territory
AG	Antigua and Barbuda	RE	Réunion (France)
AS	American Samoa (US)	KN	Saint Kitts and Nevis
AI	Anguilla (UK)	LC	Saint Lucia
AW	Aruba (Netherlands)	MF	Saint Martin (France)
BB	Barbados	WS	Samoa
BM	Bermuda (UK)	CO-SP	San Andrés and Providencia (Colombia)
BQ	Bonaire, Sint Eustatius & Saba (Netherlands)	ST	São Tomé and Príncipe
BH	Bahamas	SX	Sint Maarten (Netherlands)
HN-BI	Bay Islands (Honduras)	SC	Seychelles
BZ	Belize (Cayes)	SB	Solomon Islands
KY	Cayman Islands (UK)	VC	St. Vincent and the Grenadines
KM	Comoros	TO	Tonga
CV	Cape Verde	TT	Trinidad and Tobago
CW	Curaçao (Netherlands)	TC	Turks and Caicos
DM	Dominica	TV	Tuvalu
DO	Dominican Republic	VI	US Virgin Islands (US)
FM	Federated States of Micronesia	VU	Vanuatu
FJ	Fiji	VG	Virgin Islands (UK)
PF	French Polynesia (France)		
GD	Grenada		
GP	Guadeloupe (France)		
GU	Guam (US)		
HT	Haiti		
US-HI	Hawaii (US)		
JM	Jamaica		
KI	Kiribati		
MV	Maldives		
MH	Marshall Islands		
MQ	Martinique (France)		
MU	Mauritius		
YT	Mayotte (France)		
MP	Northern Mariana Islands (US)		
NC	New Caledonia (France)		
NR	Nauru		
PG	Papua New Guinea		
PR	Puerto Rico (US)		
PW	Palau		

4 Supplementary Figures

Supplementary Figure 2: **Perceptions of future community conditions, by country or territory.** Country/territory-level weighted average responses to the following survey question (with areas missing data from 1 or more quotas unweighted or with samples sizes $<$ 50 marked in blue): *When you imagine your community in 20 years, do you think your community will be...*

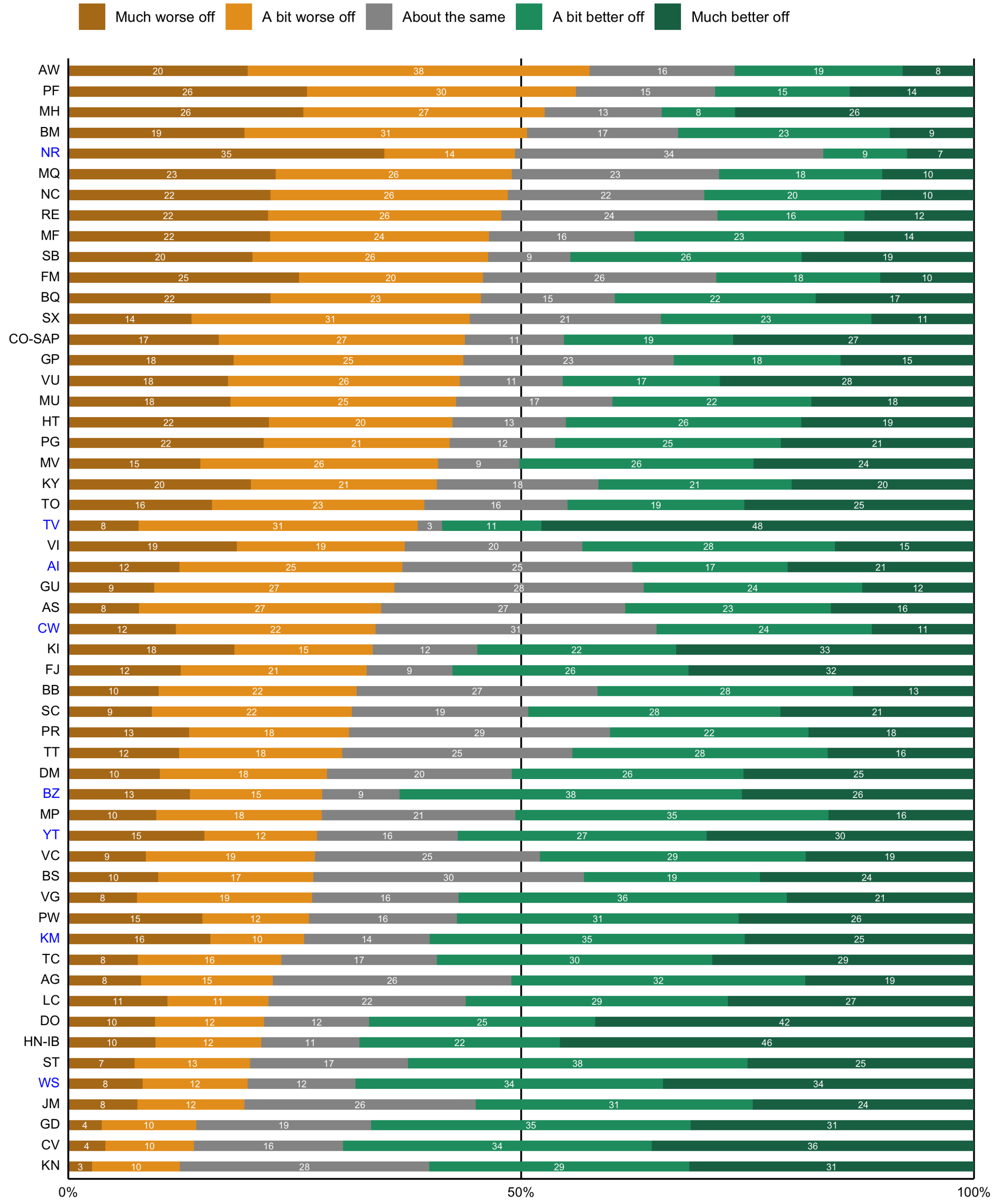

Supplementary Figure 3: **Belief in climate change**. Country/territory-level weighted average responses. Green = believe; grey = don't know; orange = don't believe

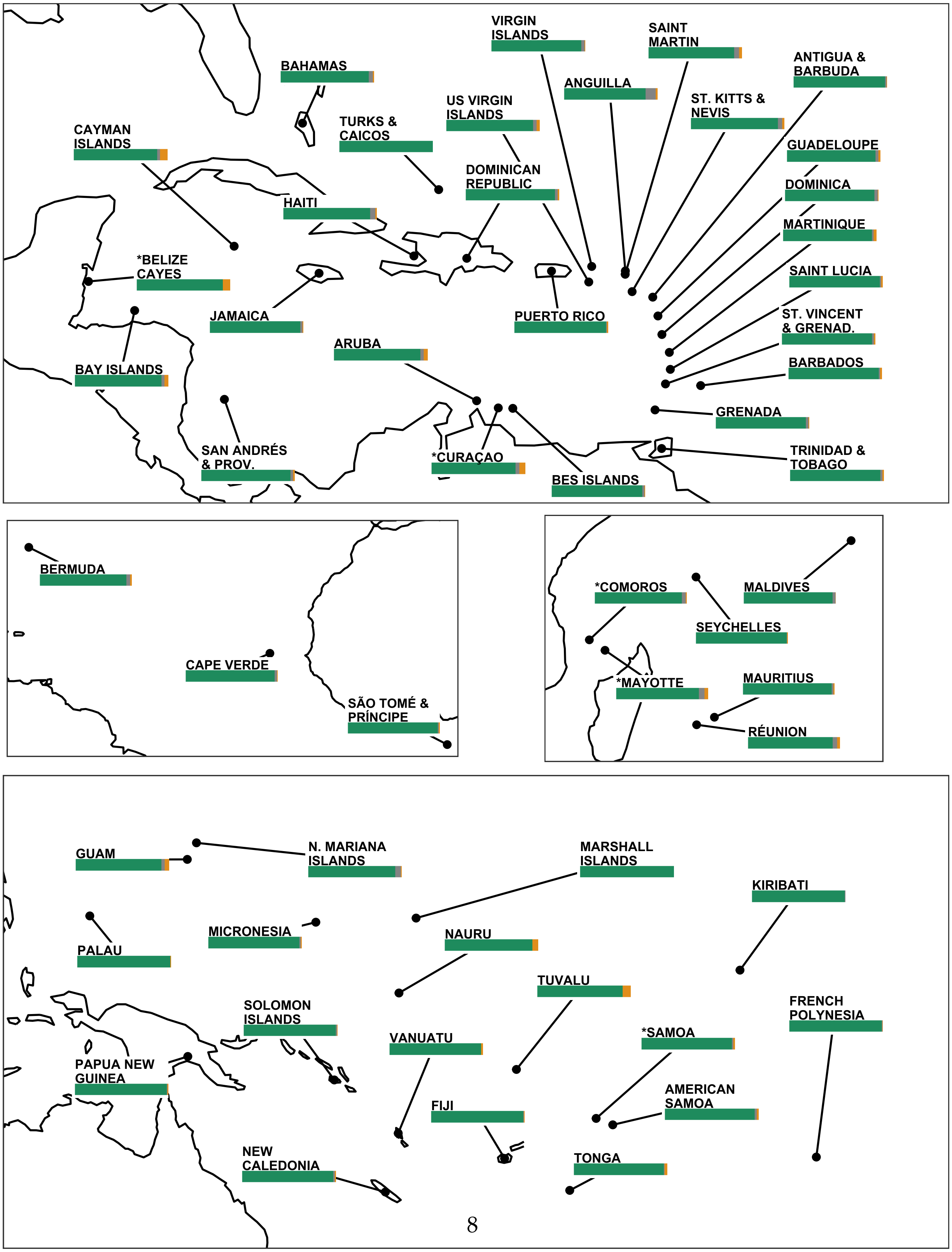

Supplementary Figure 4: **Perceived exposure to coastal vulnerability**. Country/territory-level weighted average responses to the following survey question (with areas missing data from 1 or more quotas unweighted or with samples sizes < 50 marked in blue): *How worried are you about sea-level rise or coastal erosion in your local area?*

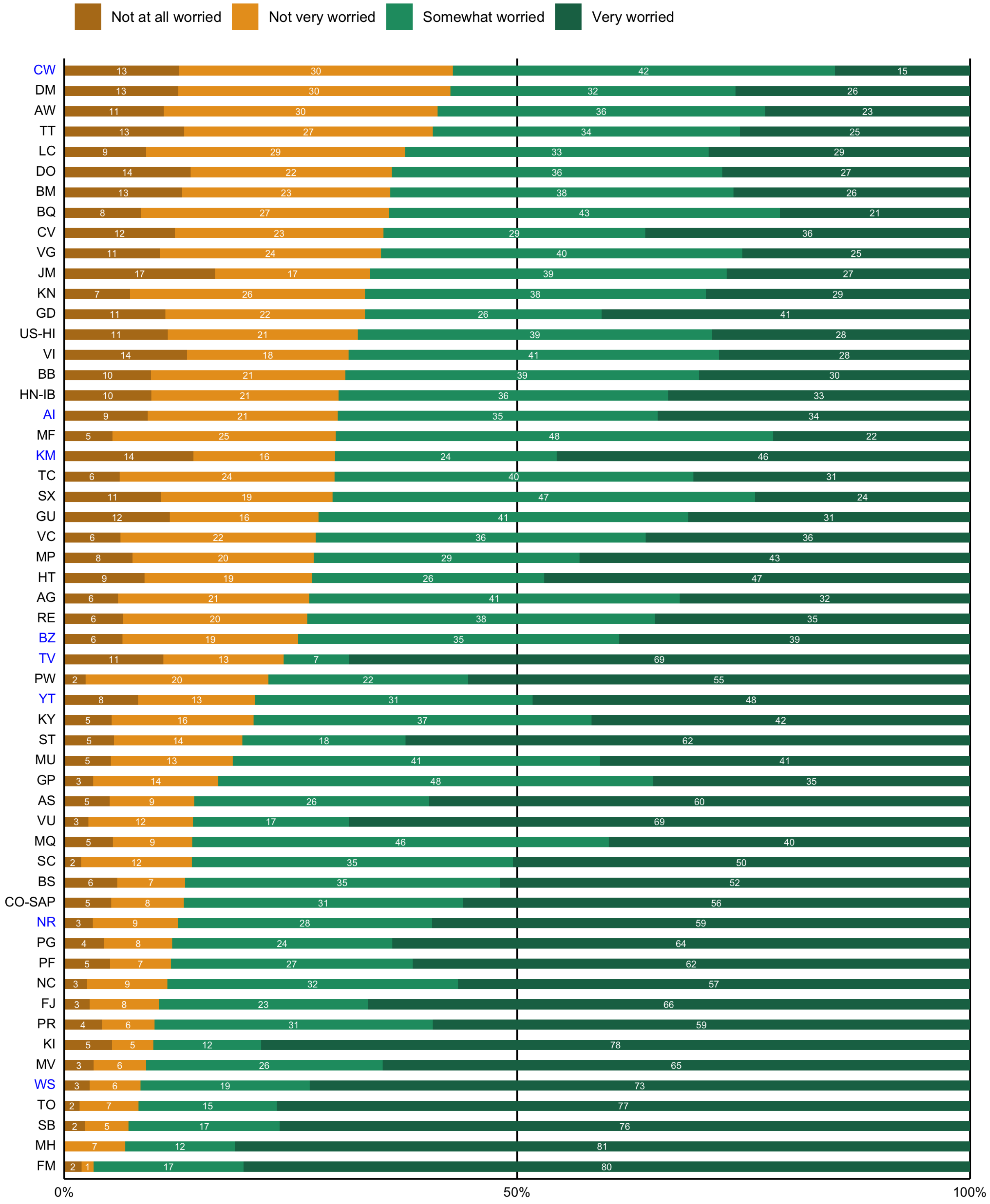

Supplementary Figure 5: **Perceived exposure to water contamination**. Country/territory-level weighted average responses to the following survey question (with areas missing data from 1 or more quotas unweighted or with samples sizes < 50 marked in blue): *How much, if at all, has sea water contaminated your drinking water?*

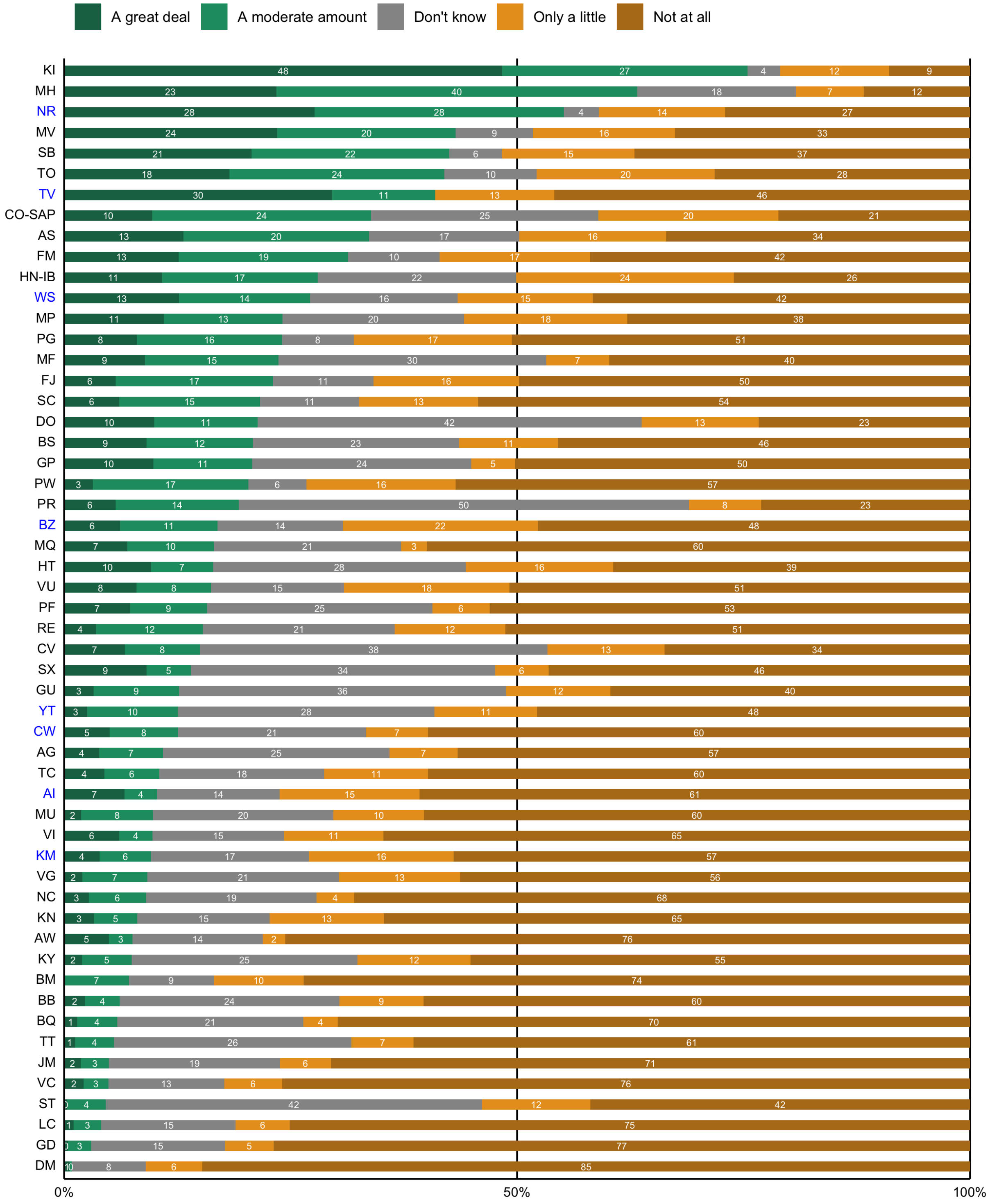

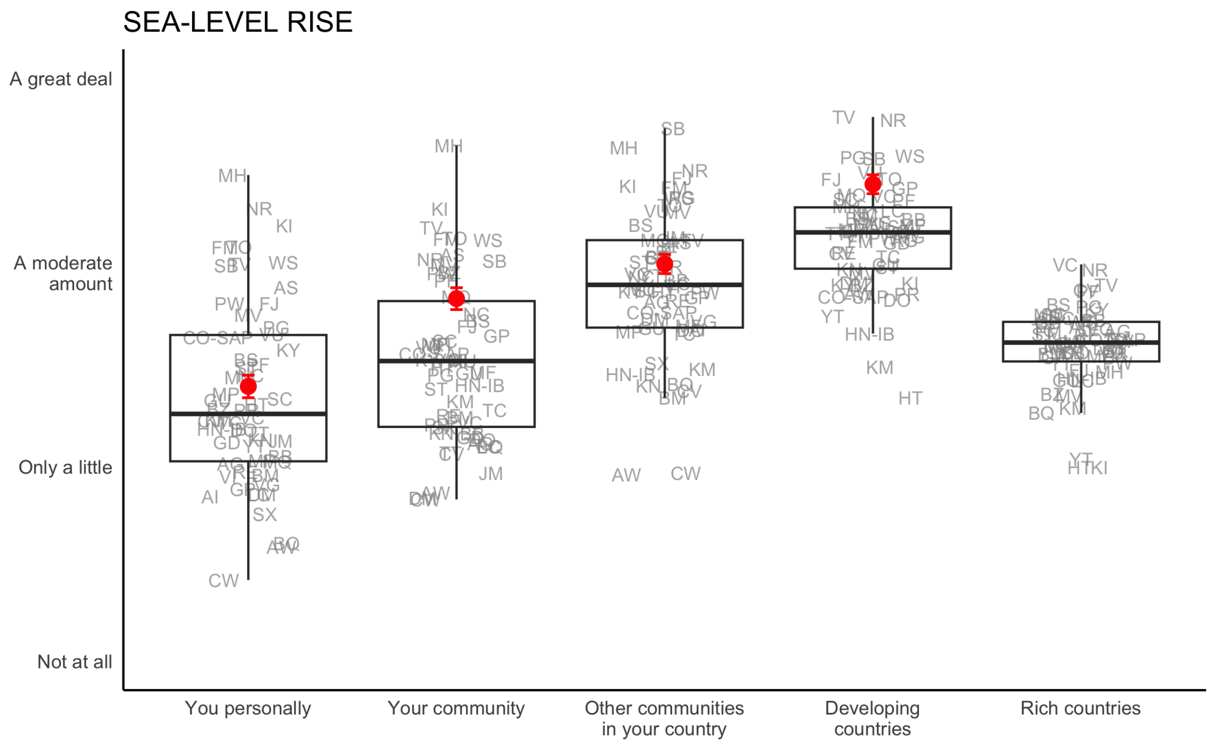

Supplementary Figure 6: **Perceived impact of sea-level rise on select groups**. The figure shows country/territory-level weighted average responses to the following survey question (with areas missing data from 1 or more quotas unweighted or with samples sizes < 50 marked in blue): *How much do you think the following people or groups experienced the impacts of sea-level rise up to this point?*. To avoid survey fatigue, respondents were randomly assigned to either receive the sea-level rise or the climate change impact question. Y-axis is a scale from 1 (Not at all) to 4 (A great deal). Random jitter has been applied within each column for visual assistance. Points in red show the average response for the US

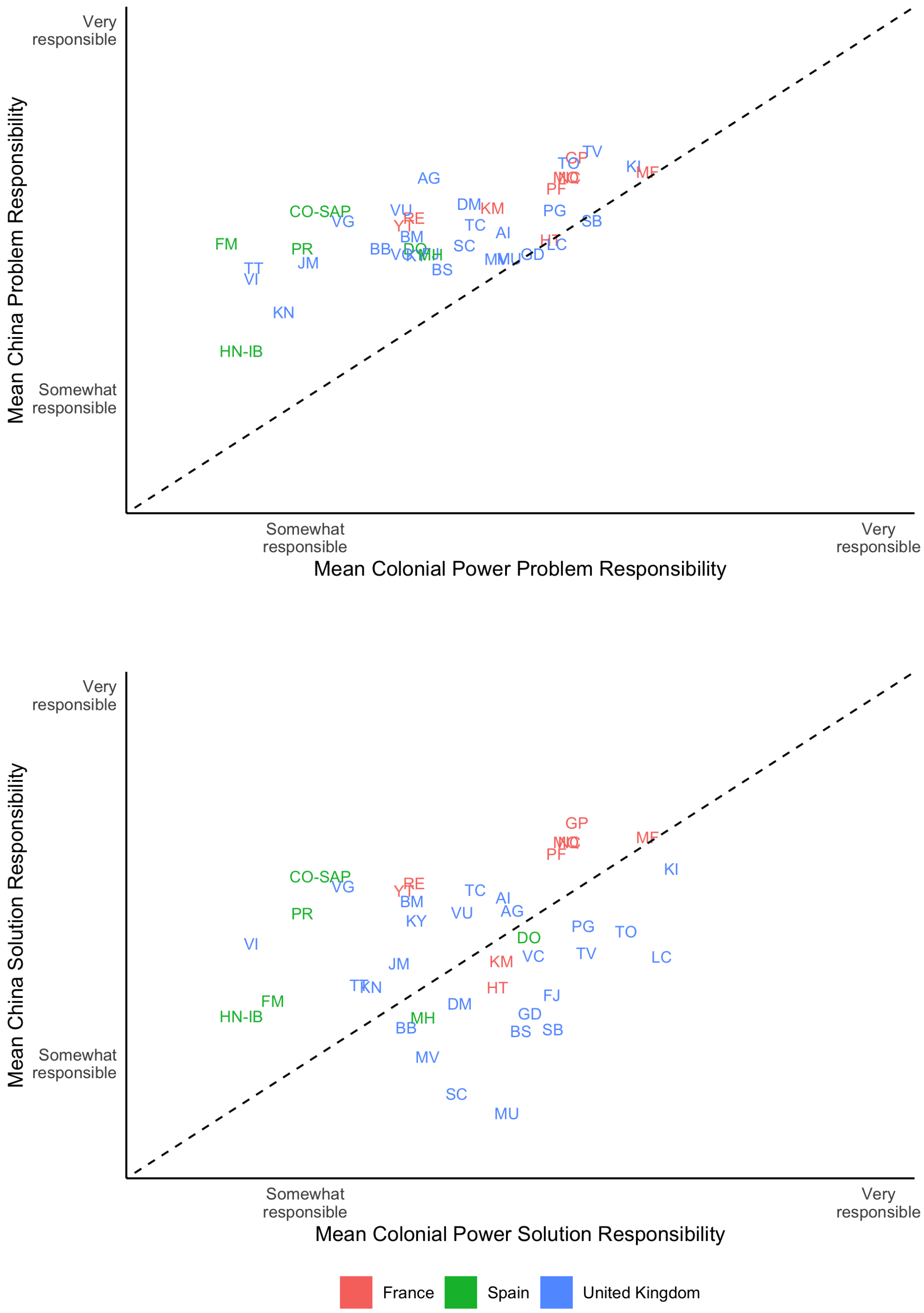

Supplementary Figure 7: **Attribution of responsibility of China vs. select colonial powers**. Top pane plots mean perception of the China being responsible for climate change vs. a country or territory's colonial power. Bottom pane plots mean perceptions of either the China or colonial power being responsible for solving climate change. Country/territory means are weighted.

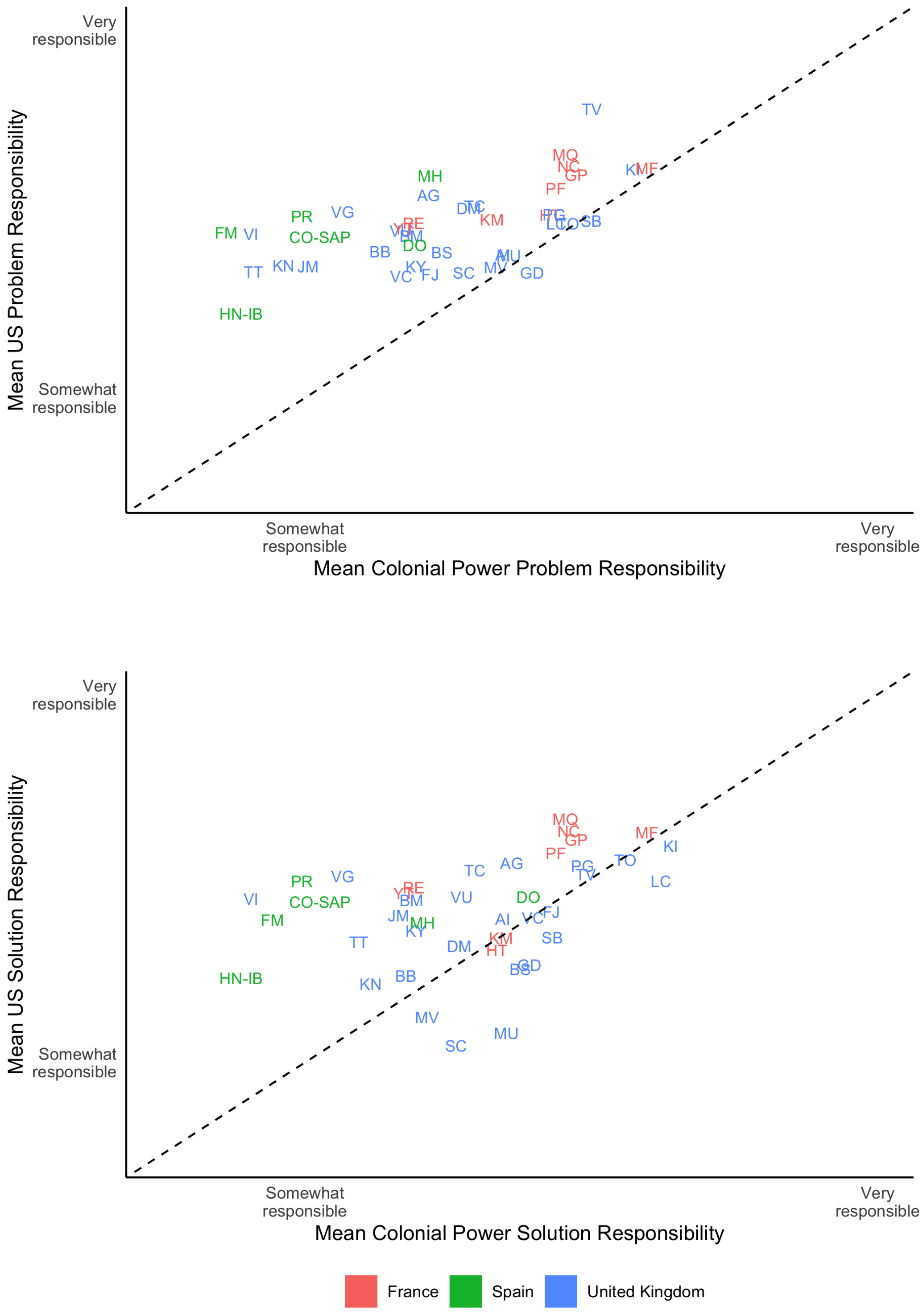

Supplementary Figure 8: **Attribution of responsibility of US vs. select colonial powers**. Top pane plots mean perception of the US being responsible for climate change vs. a country or territory's colonial power. Bottom pane plots mean perceptions of either the US or colonial power being responsible for solving climate change. Country/territory means are weighted.

Supplementary Table 3 complements the descriptive Figure 3 in the main text. To quantify the extent to which survey respondents attribute differential responsibility for causing and addressing climate change, we regressed attribution of responsibility on indicators for each country about which respondents were asked about responsibility for causing and addressing climate change. The regression can be interpreted as a difference in mean attribution of responsibility across the countries included in the question. Relative to the US (the omitted category, representing a high-emitting country), individuals believe that their colonial powers are somewhat less responsible for causing climate change (a difference of 0.24 points on a 4-point Likert scale). They also attribute less responsibility for addressing climate change to their colonial powers, but this difference is half the size as the difference in causal attribution. When examining the data in terms of average responses, the relative parity in attribution of responsibility for addressing climate change appears even stronger. Average attribution of responsibility to the US and the colonial power is 3.4 and 3.3, respectively, corresponding to an average value of "somewhat responsible." Notably, even though individuals view their own countries as less responsible for causing climate change (a difference of 0.72 Likert scale points, average value of 2.7), they nonetheless believe their countries should also be held "somewhat responsible" for solutions (an average value of 2.9 on the 4-point Likert scale).

	Cause	Address
(Intercept)	3.42^{***}	3.38^{***}
	(0.01)	(0.01)
China	-0.00	-0.11^{***}
	(0.01)	(0.01)
Colonial Power	-0.24^{***}	-0.12^{***}
	(0.01)	(0.01)
Home country	-0.72^{***}	-0.44^{***}
	(0.01)	(0.01)
Regional power	-0.38^{***}	-0.29^{***}
	(0.01)	(0.01)
Saudi Arabia	-0.35^{***}	-0.29^{***}
	(0.01)	(0.01)
R^2	0.07	0.02
Adj. R^2	0.07	0.02

$^{***}p < 0.001$; $^{**}p < 0.01$; $^{*}p < 0.05$

Supplementary Table 3: **Difference in means test for attribution of responsibility for causing and addressing climate change:** Results are based on a survey question asking how much each of the countries shown was responsible for causing (Column 1) and addressing (Column 2) climate change. We regressed attribution of responsibility (4-point Likert scales, standardized to have mean of zero and standard deviation of one) on indicators for each country included in the survey question. The US is the omitted category. Standard errors are clustered by respondent.

	Low Concern about Extreme Weather
age	-0.001^{***}
	(0.0003)
male	0.047^{***}
	(0.007)
not indigenous	0.035^{***}
	(0.013)
has child	-0.048
	(0.061)
homeowner	0.024
	(0.033)
wealth	0.013^{***}
	(0.002)
home directly on ocean	0.015
	(0.057)
home medium distance from ocena	-0.012
	(0.055)
home short distance from ocean	-0.014
	(0.055)
home not close to ocean	0.010
	(0.056)
elevation:high	-0.014
	(0.023)
elevation:low	-0.025
	(0.023)
Constant	0.151^{**}
	(0.076)
Observations	13,096
R^2	0.017
Adjusted R^2	0.015
Residual Std. Error	0.363 (df = 13078)
F Statistic	13.029^{***} (df = 17; 13078)
Note:	*p<0.1; **p<0.05; ***p<0.01

Supplementary Table 4: **Predicting which respondents view extreme weather as something that will impact their country "only a little" or "not at all":** DV is a recoded version of the survey question presented in the manuscript's Figure 1, with a dichotomous variable taking a value of 1 if the respondent answered "only a little" or "not at all" and 0 otherwise. Attitudinal variables (e.g. belief in anthropogenic climate change) are not used as predictors given endogeneity concerns, but results should be viewed as exploratory since they are not causally identified.

Supplementary Figure 9: **Average desired contributions from each country, for respondents in the control group.** The figure shows average responses to our question about how much each country should contribute to the global adaptation fund. Each individual was asked to state their desired contribution from a randomly assigned country. Each individual was also randomly assigned to receive no information about that country's emissions or to receive information about the country's cumulative and/or current emissions. Responses are shown only for those individuals who did not receive any information about cumulative or current emissions.

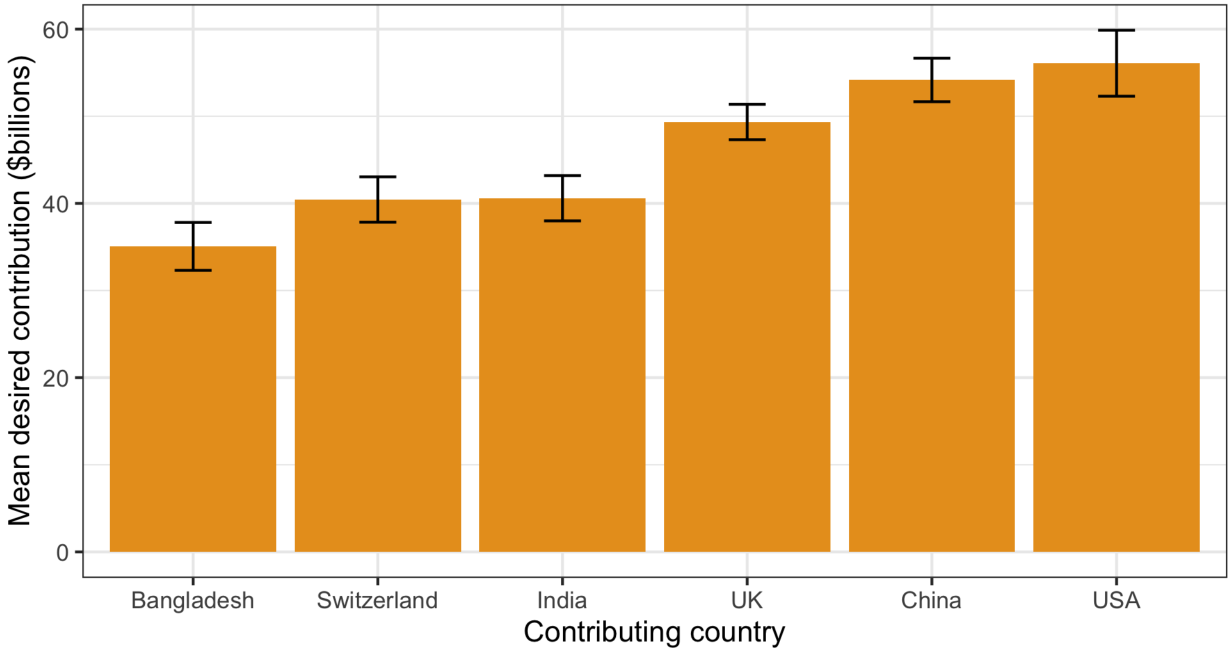

Figure 9 complements Figure 4 in the main text by showing the amount that individuals would like each country to contribute to the global adaptation fund, if they do not receive any information about current or cumulative emissions from that country. The figure shows that, without any information about emissions, people assign a higher responsibility for contributing to the fund to the US, China and, to a slightly lesser extent, the UK. By contrast, at baseline, individuals do not expect as strong a contribution from Switzerland (despite its wealth), India (despite its current emissions), or Bangladesh.

5 Geographic quotas across sampled countries and territories

Supplementary Table 5: **Geographic quotas for countries and territories in sample**

Country Territory	Quota	Geographic Coverage
Antigua and Barbuda	none	
Anguilla (UK)	none	
American Samoa (US)	none	
Aruba (Netherlands)	none	
Barbados	capital	St. Michael parish
	rest	All other parishes
Bermuda (UK)	none	
Bonaire, Sint Eustatius and Saba (Netherlands)	none	
Bahamas	capital	Island of New Providence
	rest	All other districts
Belize (Cayes)[1]	none	
San Andrés and Providencia (Colombia)	main	San Andrés
	rest	Rest of department
Cape Verde[2]	barlavento	Barlavento islands
	sotavento	Sotavento islands
Curaçao (Netherlands)	capital	City of Willemstad
	rest	Rest of Curaçao
Dominica	none	
Dominican Republic	capital	Provinces of Distrito Nacional and Santo Domingo
	urban	Provinces with pop. density $> 200/\text{km}^2$
	rural	Provinces with pop. density $\leq 200/\text{km}^2$
Fiji	main	Provinces of Ba and Rewa
	westcentral	Provinces of Natasiri, Namosi, Serua, Tailevu, Nadroga-Navosa, Ra

[1]Separate ad sets were used for San Pedro/Ambergris Caye, and Caye Caulker
[2]Separate ad sets were used for Praia vs. the remainder of the Sotavento island group

	eastnorth	Provinces of Bua, Cakaudrove, Macuata, Kadavu, Lau, Lomaiviti, Rotuma
Federated States of Micronesia	none	
Grenada	none	
Guadeloupe (France)	main	Pointe-à-Pitre-Les Abymes metro area
	rest	Rest of Guadeloupe
Guam (US)	none	
Bay Islands (Honduras)	none	
Haiti	portauprince	Dép. of Ouest
	artibonite	Dép. of Artibonite
	north	Déps. of Centre, Nord, Nord-Est, Nord-Ouest
	south	Déps. of Grand'Anse, Nippes, Sud, Sud-Est
Jamaica	main	Parishes of Kingston, St. Andrew, St. Catherine
	rest	All other parishes
Kiribati	none	
Comoros	grandecomore	Island of Grande Comore (Ngazidja)
	anjouan	Island of Anjouan (Ndzwani)
	moheli	Island of Mohéli (Mwali)
Saint Kitts and Nevis	none	
Cayman Islands (UK)	none	
Saint Lucia	main	District of Castries
	rest	All other districts
Saint Martin (France)	none	
Marshall Islands	none	
Northern Mariana Islands (US)	none	
Martinique (France)	main	Communes of Fort-de-France, Le Lamentin, Saint-Joseph, Schœlcher
	rest	All other communes
Mauritius	main	Districts of Port Louis, Plaines Wilhems
Maldives	male	City of Malé
	rest	Rest of Maldives

New Caledonia (France)	main	Communes of Dumbéa and Nouméa
	rest	Rest of New Caledonia
Nauru	none	
French Polynesia (France)	main	Island of Tahiti
	rest	Rest of French Polynesia
Papua New Guinea	capital	National Capital District (Port Moresby)
	highlands	Provinces of Southern Highlands, Western Highlands, Enga, Chimbu, Eastern Highlands, Hela and Jiwaka
	islands	Provinces of Manus, New Ireland, West New Britain, Bougainville and East New Britain
	momase	Provinces of East Sepik, West Sepik, Madang and Morobe
	southern	Provinces of Milne Bay, Northern, Central, Gulf and Western
Puerto Rico (US)	sanjuan	Municipalities of Bayamon, Carolina, Catano, Guaynabo, San Juan, and Trujillo Alto
	cities	Aguadilla, Arecibo, Caguas, Humacao, Mayaguez, Ponce, Toa Alta, Toa Baja, Vega Baja
	rest	All other municipalities
Palau	none	
Réunion (France)	benoitdenis	Arrs. of Saint-Benoit, Saint-Denis
	paul	Arr. of Saint-Paul
	pierre	Arr. of Saint-Pierre
Solomon Islands	honiara	Honiara province
	rest	All other provinces
Seychelles	main	Agua Grande district
	rest	All other districts
São Tomé and Príncipe	none	
Sint Maarten (NL)	none	

Turks and Caicos	none	
Tonga	none	
Trinidad and Tobago	high	Regions of Port of Spain, San Fernando, Chaguanas, Arima
	med	Regions of Point Fortin, Diego Martin, Penal–Debe, San Juan–Laventille, Tunapuna–Piarce
	low	Regions of Couva–Tabaquite–Talparo, Mayaro–Rio Claro, Princes Town, Sangre Grande, Siparia
	tobago	Tobago region
Tuvalu	none	
Hawaii (US)	bigisland	
	kauai	Kauai county
	maui	Maui and Kalawao counties
	bigisland	Hawai'i county
	oahu	Honolulu county
St. Vincent and the Gren.	none	
Virgin Islands (UK)	none	
US Virgin Islands	none	
Vanuatu	none	
Samoa	none	
Mayotte (France)	mamoudzou	Commune of Mamoudzou
	pamandzi	Communes of Pamandzi and Dzaoudzi
	rest	All other communes

6 Facebook Sampling Costs

Supplementary Table 6: **Facebook ad cost and reach statistics by target geography**

Country	Total Spend	Conversions	Link Clicks	Reach	Impressions
Antigua & Barbuda	$479.66	165	1,423	38,568	222,765
Anguilla	$614.76	41	853	8,276	96,626
American Samoa	$537.99	141	1,707	22,265	266,292
Aruba	$247.98	157	925	27,696	89,249
Barbados	$952.26	306	2,205	87,120	310,528
Bermuda	$764.88	128	1,266	19,044	143,982
Bonaire-Sint Eustatius-Saba	$382.27	82	731	12,216	108,394
Bahamas	$900.00	392	3,531	83,541	368,045
Belize (Cayes)	$214.34	39	799	14,236	128,202
San Andrés and Providencia	$551.07	230	3,680	28,424[3]	113,132
Cape Verde	$318.78	618	6,143	106,784	423,401
Curaçao	$295.50	176	1,108	31,800	85,309
Dominica	$489.90	191	1,672	30,014	249,088
Dominican Republic	$396.80	906	5,101	155,328	243,189
Fiji	$867.10	1,366	7,746	230,880	958,600
Micronesia	$793.23	143	2,614	32,176	413,484
Grenada	$250.00	170	1,340	37,640	154,948
Guadeloupe	$941.64	127	1,868	56,544	227,336
Guam	$649.13	229	1,709	45,384	232,405
Bay Islands (Honduras)	$187.20	171	1,707	24,896	69,217
Haiti	$1341.64	774	20,386	456,708	1,664,415
Jamaica	$683.88	679	4,110	161,751	351,614
Kiribati	$1046.37	127	3,850	43,752	622,516
Comoros	$1295.66	299	11,666	157,056	1,510,563
Saint Kitts and Nevis	$408.56	117	1,144	23,176	136,363
Cayman Islands	$650.00	131	1,301	26,672	143,179
Saint Lucia	$672.04	314	2,553	58,678	304,824
Saint Martin (France)	$783.58	40	1,070	13,140	190,165

[3]Estimated only, lower bound.

Marshall Islands	$655.09	65	1,776	17,744	268,979
Northern Mariana Islands	$430.85	141	1,340	19,166	113,781
Martinique	$691.68	141	1,588	52,096	179,158
Mauritius	$690.19	1,065	6,318	150,625	428,272
Maldives	$1648.00	336	6,013	108,625	1,201,628
New Caledonia	$697.75	238	2,135	75,363	214,930
Nauru	$528.56	35	740	7,461	115,118
French Polynesia	$950.00	275	3,114	80,323	381,262
Papua New Guinea	$1338.14	703	10,126	321,163	1,609,449
Puerto Rico	$730.92	706	2,722	58,431[4]	149,395
Palau	$336.12	83	771	11,740	108,987
Réunion	$1114.37	177	2,097	116,939	440,198
Solomon Islands	$981.03	345	5,160	751,110	77,888
Seychelles	$711.32	177	3,193	437,212	53,475
São Tomé and Príncipe	$729.01	156	3,133	345,410	40,599
Sint Maarten (NL)	$1085.00	69	1,654	336,635	24,568
Turks and Caicos	$1250.00	77	2,343	406,698	22,744
Tonga	$476.91	180	2,254	439,829	47,137
Trinidad and Tobago	$958.02	977	5,554	536,697	184,543
Tuvalu	$624.96	25	720	130,058	5,722
Hawaii (US)	$1431.53	333	1,251	69,721	35,896
St. Vincent and the Gren.	$228.97	107	1,140	124,809	35,392
Virgin Islands (UK)	$470.53	73	931	139,780	13,996
US Virgin Islands	$874.63	134	944	153,655	14,876
Vanuatu	$887.47	147	2,687	483,391	64,496
Samoa	$534.07	154	2,643	547,367	73,315
Mayotte (France)	$1050.00	75	2,639	553,860	60,008

[4] Estimated lower bound

7 Sample Sizes by Survey Block

Supplementary Table 7: **Sample sizes by survey block**

**Country**	**Block 1**	**Block 2**	**Block 3**	**Block 4**	**Block 5**
Total	25931	24783	23365	22082	20026
Antigua & Barbuda	251	248	230	215	192
Anguilla	96	94	85	75	61
American Samoa	258	253	236	217	190
Aruba	253	243	224	201	180
Barbados	487	461	440	411	370
Bay Islands (Honduras)	344	320	301	267	225
Bermuda	198	193	181	167	159
Bahamas	646	613	581	547	484
Bonaire-Sint Eustatius-Saba	149	144	128	115	105
Belize (Cayes)	73	67	63	61	50
Cape Verde	987	954	890	829	752
Cayman Islands	227	213	193	182	168
Comoros	626	586	539	504	454
Curaçao	302	286	266	250	217
Dominica	293	279	262	246	219
Dominican Republic	1427	1375	1295	1227	1091
Fiji	1937	1870	1803	1737	1599
French Polynesia	448	435	409	388	351
Grenada	298	273	254	228	205
Guadeloupe	270	258	238	227	194
Guam	354	341	326	309	282
Haiti	1337	1279	1120	1162	1058
Hawaii (US)	448	427	410	398	379
Jamaica	1122	1060	989	928	815
Kiribati	209	197	184	172	155
Marshall Islands	107	100	91	88	83
Northern Mariana Islands	221	218	207	199	180
Martinique	268	258	247	235	209
Mauritius	1606	1529	1460	1391	1276

Maldives	556	518	488	450	408
Mayotte	174	166	148	137	116
Micronesia	204	196	185	179	173
New Caledonia	378	364	344	328	299
Nauru	46	42	41	40	39
Papua New Guinea	1209	1169	1099	1049	974
Puerto Rico	968	940	901	877	821
Palau	111	105	103	101	94
Réunion	306	297	274	263	236
Saint Kitts and Nevis	212	190	175	167	152
Saint Lucia	503	476	445	413	365
Saint Martin (France)	104	98	90	79	70
Samoa	233	229	213	205	189
San Andrés and Providencia	427	381	347	321	294
São Tomé and Príncipe	265	258	240	218	206
Seychelles	302	285	261	247	221
Sint Maarten (NL)	121	111	104	101	91
Solomon Islands	505	488	467	447	421
St. Vincent and the Gren.	353	330	311	278	247
Tonga	241	236	230	218	211
Trinidad and Tobago	1620	1560	1477	1386	1256
Turks and Caicos	158	151	144	133	109
Tuvalu	44	44	42	39	35
US Virgin Islands	186	183	178	171	162
Vanuatu	235	225	211	206	190
Virgin Islands (UK)	123	118	109	101	87

8 Details on geographic quotas for countries and territories in sample

Here we present detailed tables that provide our final sample sizes by quota and level of survey completion. The leftmost column of each table provides the quotas for each country or territory. These are age by gender for less populous countries and territories. They are age by gender by region for more populous countries and territories (see Sampling Frame above). The geographic

extent of these regional quotas is defined in the captions. The second column "Target" provides the target n established during the sampling process for each quota. The survey blocks are defined below in the section Survey Instrument.[5]

"Block 1" through "Block 5" provide the number of respondents who answered any questions in each of these successive survey blocks. As described above (see Survey Instrument): Block 1 addressed topics of migration generally, Block 2 addressed experiences with extreme weather, Block 3 addressed climate change attitudes, Block 4 asked questions about climate adaptation experiences, and Block 5 asked questions about global climate politics and finance. The final column "Finished" records the number of individuals who clicked through to the end of the survey (including the final demographic bank) and formally submitted their answers. A small number of individuals clicked through the entire survey to register for the prize drawing without answering many (if any) questions. These individuals are included in the Finished column even if they are not included in counts for specific blocks for which they did not answer questions.

[5]In these SI tables, we also include captions that annotate country or territory-specific sampling details that condition how analysis is conducted of those countries. This includes descriptions of missing data, the number of respondents (in the "Block 1" column) who self-reported living in a country but entered the survey through a different country or territory's Facebook ad and, for small countries where 0.25% of the adult population is less than 150 individuals, the n associated with this threshold that was used during sampling as a benchmark target for total sample size (see Sampling Process).

9 Survey Instrument

The survey instrument is reproduced here in its complete form, with questions listed in the presented survey order. All questions analyzed in the paper and/or necessary to replicate paper and SI results are included in the replication archive. Other questions are part of separate papers under review or preparation. Please inquire at mildenberger@ucsb.edu for access to these questions in advance of the public deposition of all analyzed and unanalyzed data.

`num_FB_surveys`: Before we start, we are curious to know: how many other public opinion surveys have you taken through Facebook in the past month?

- 0
- 1
- 2
- 3 or more

`community_feeling`: Please tell us how much you agree or disagree with the following statement: **My community means a lot to me.**

- Strongly agree
- Somewhat agree
- Neither agree nor disagree
- Somewhat disagree
- Strongly disagree

`future_scale`: When you imagine your community in 20 years, do you think your community will be better off, about the same, or worse off?

- Much better off
- A bit better off
- About the same
- A bit worse off
- Much worse off

Those who answered ″Much better off″ or "A bit better off" in the previous question are asked the following question:

`future_open_good`: Using just a few words: When you imagine your community in 20 years, what is the most important reason you think your community will be better off?

Those who answered ″A bit worse off″ or "Much worse off" to the future_scale question are asked the following question

---

`future_open_bad`: Using just a few words: When you imagine your community in 20 years, what is the most important reason you think your community will be worse off?

Display this question to those where a territory is not indicated:

`move_family`: Up until now, has **anyone in your family or a close friend** temporarily or permanently moved to another country?

- Yes
- No

`pastexperience`: In the **past five years**, have you personally experienced any of the following? [select all that apply]

- Hurricane, typhoon or cyclone
- Wildfire
- Extreme Heat
- Drought
- Severe storm
- Flood
- Landslide, mudslide, or avalanche
- Fish die-off or dead sea animals washed ashore (display if version = ”arctic”)
- Permafrost thaw (display if version = ”arctic”)
- Early season ice melt (display if version = ”arctic”)
- Glacial retreat (display if version = ”arctic”)
- None of the above

`futureexperience`: How much do you think extreme weather (like extreme heat, drought, severe storms, floods, hurricanes, or wildfires) will harm people in your community in the next five years?

- Not at all
- Only a little
- A moderate amount

- A great deal
- Don't know

---

slr_water: How much, if at all, has sea water contaminated your drinking water?

- Not at all
- Only a little
- A moderate amount
- A great deal
- Don't know

Display this question if slr_or_climate = slr:

---

slr_impacts: The following questions ask about sea-level rise and coastal erosion. Sea-level rise means an increase in the water level of the oceans. Coastal erosion means the loss of land along coastlines from sea level rise, storms, and flooding.
How much do you think the following people or groups experienced the impacts of sea-level rise or coastal erosion up to this point?

	Not at all	Only a little	A moderate amount	A great deal	Don't know
You personally	o	o	o	o	o
Your community	o	o	o	o	o
Other communities in `lowercountry`	o	o	o	o	o
Developing countries	o	o	o	o	o
Rich countries	o	o	o	o	o

`slr_worry`: How worried are you about sea-level rise or coastal erosion in your local area?

- Very worried
- Somewhat worried
- Not very worried
- Not at all worried

`gw_happen`: Recently, you may have noticed that climate change has been getting some attention in the news. Climate change is also sometimes called global warming. Climate change refers to

the idea that the world's average temperature has been increasing over the past 150 years, may be increasing more in the future, and that the world's climate may change as a result.

Do you think that climate change is happening?

- Yes
- No
- Don't Know

`gw_lloyds`: Do you think that climate change is a very serious threat, a somewhat serious threat, or not a threat at all to the people in this country in the next 20 years? If you don't know, please just say so.

- Very serious threat
- Somewhat serious threat
- Not a threat at all
- I don't know

Display this question if `slr_or_climate` = climate:

`climate_impacts`: How much do you think the following people or groups experienced the impacts of climate change up to this point?

	Not at all	Only a little	A moderate amount	A great deal	Don't know
You personally	o	o	o	o	o
Your community	o	o	o	o	o
Other communities in `lowercountry`	o	o	o	o	o
Developing countries	o	o	o	o	o
Rich countries	o	o	o	o	o

`storms_climate`: How much do you agree or disagree with the following statement:

Storms in my community have been getting worse because of climate change.

- Strongly agree
- Somewhat agree
- Neither agree or disagree
- Somewhat disagree
- Strongly disagree

`slr_climate`: How much do you agree or disagree with the following statement:

Coastal erosion, flooding and sea-level rise in my community have been getting worse because of climate change.

- Strongly agree
- Somewhat agree
- Neither agree or disagree
- Somewhat disagree
- Strongly disagree

`mitigation`: How much do you agree or disagree with the following statement:

My country should transition to 100% clean energy, like wind and solar, by 2035, even if it slightly increases the costs of electricity in the short-term.

- Strongly agree
- Somewhat agree
- Neither agree or disagree
- Somewhat disagree
- Strongly disagree

`adapt_benefits`: Have you experienced any of the following measures to **manage your exposure to sea level rise, coastal erosion, or storms/cyclones?** Please select all that apply:

- Building projects to protect the coast (for example seawalls and levees)
- Planning in advance for emergencies

- Compensation for people who are impacted

Display this question if 1 or more choices were selected in the previous question, Display each choice only if previously indicated:

`adapt_responsible`: Who do you think is the main group responsible for doing the thing(s) you selected?

	Your government	A community association	A foreign entity (including an international organization)	Other
Building projects to protect the coast (for example seawalls and levees)	o	o	o	o
Planning in advance for emergencies	o	o	o	o
Compensation for people who are impacted	o	o	o	o

adapt_intro:We will now provide you with a hypothetical example of a community project. People have different opinions about this issue and there are no right or wrong answers. Please take your time when reading the prompts.

Rising sea levels have led to coastal erosion, floods, and irreparable damage to coastal communities. **In response, adapt_actor is adapt_activity in your community.**

`adapt_support`: Based on the information provided, how strongly do you support or oppose `adapt_activity` in your community?

- Support strongly
- Support somewhat
- Oppose somewhat
- Oppose strongly

`adapt_effective`: Would you say that `adapt_activity` will protect your community from rising sea levels?

- Yes, strongly
- Yes, somewhat
- No, somewhat
- No, strongly

Display this question if `adapt_activity` does not equal planting mangroves to reduce the impacts of floods and sea level rise

---

`adapt_living`: Would you say that `adapt_activity` will improve your personal living conditions?

- Yes, strongly
- Yes, somewhat
- No, somewhat

- No, strongly

Display this question if `adapt_activity` does not equal planting mangroves to reduce the impacts of floods and sea level rise

---

`adapt_political`: Again, still assuming that `adapt_actor` is responsible for `adapt_activity`, how strongly do you agree or disagree with each of the following statements:

	Strongly agree	Somewhat agree	Neither agree nor disagree	Somewhat disagree	Strongly disagree
I would be more likely to trust my government to provide services to my community	o	o	o	o	o
I would be more likely to get involved in community activities	o	o	o	o	o
I would be more likely to vote in elections	o	o	o	o	o

Display this question to those where a territory is not indicated:

---

responsible_cause: When you think of climate change and its impacts on your country, **how responsible do you think each of the following countries is for causing the problem of climate change?**

	Very responsible	Somewhat responsible	Not very responsible	Not at all responsible
US	o	o	o	o
China	o	o	o	o
Saudi Arabia	o	o	o	o
capcountry	o	o	o	o
reg	o	o	o	o
col	o	o	o	o

Display this question to those where a territory is indicated:

---

cause_ter: When you think of climate change and its impacts on your territory, **how responsible do you think each of the following countries or territories is for causing the problem of climate change?**

https://www.overleaf.com/project/62ca57323dafae726345a871

	Very responsible	Somewhat responsible	Not very responsible	Not at all responsible
US	o	o	o	o
China	o	o	o	o
Saudi Arabia	o	o	o	o
`capcountry`	o	o	o	o
`reg`	o	o	o	o
`col`	o	o	o	o

Display this question to those where a territory is not indicated:

`responsible_solve`: When you think of climate change and its impacts on your country, **how responsible do you think each of the following countries is for finding a solution to the problem of climate change?**

	Very responsible	Somewhat responsible	Not very responsible	Not at all responsible
US	o	o	o	o
China	o	o	o	o
Saudi Arabia	o	o	o	o
`capcountry`	o	o	o	o
`reg`	o	o	o	o
`col`	o	o	o	o

Display this question to those where a territory is indicated:

`solve_ter`: When you think of climate change and its impacts on your territory, **how responsible do you think each of the following countries or territories is for finding a solution to the problem of climate change?**

	Very responsible	Somewhat responsible	Not very responsible	Not at all responsible
US	o	o	o	o
China	o	o	o	o
Saudi Arabia	o	o	o	o
`capcountry`	o	o	o	o
`reg`	o	o	o	o
`col`	o	o	o	o

`edu`: What is your highest level of education?

- No formal schooling
- Informal schooling
- Some primary schooling
- Primary school completed
- Intermediate school or some secondary school / high school
- Secondary school / high school completed
- Post-secondary school other than university

- Some university
- University completed
- Post-graduate

`wealthcomp`: Compared to all of the people in your country, please indicate how wealthy you consider your household to be:

- Sliding scale from 0 to 10 Least wealthy to Most wealthy

Display this question to those where a territory is not indicated:

`remittances`: Sometimes people receive money from friends and family **living in other countries** to help meet their needs. How important is such income to your quality of life?

- Very Important
- Important
- Moderately Important
- Slightly Important
- Not Important
- Don't receive money

Display this question to those where a territory is indicated:

`remittances_ter`: Sometimes people receive money from friends and family **living outside of your territory** to help meet their needs. How important is such income to your quality of life?

- Very Important
- Important
- Moderately Important
- Slightly Important
- Not Important
- Don't receive money

`ownhome`: Do you own your home?

- Yes
- No

`ocean_dist`: How far is your home from the ocean?

- Directly on the ocean
- Within a short distance of the ocean
- A medium distance away from the ocean
- I don't think of my home as close to the ocean

`ground_level`: Would you say your home is on high ground or low ground?

- High ground
- Low ground

`employ`: What is your current employment status?

- Employed full-time
- Employed part-time
- Occasionally employed
- Unemployed, and looking for work
- Unemployed, and not looking for work
- Housewife / homemaker
- Retired
- Disabled

Those who selected ”Employed full-time”, ”Employed part-time”, or ”Occasionally employed” are asked the following question:

`occupation`: Which of these occupations most closely matches your main occupation?

- Student
- Agriculture / Farming
- Fishing
- Retail worker / Vendor
- Domestic labourer
- Tradesperson (e.g. construction, electrician, mechanic)
- Factory worker

- Office worker
- Security services
- Military worker
- Education worker
- Health care worker
- Communications & Information Technology worker
- Government official
- Other (please specify)

Those who selected ”Unemployed, and looking for work”, ”Unemployed, and not looking for work”, ”Housewife / homemaker”, ”Retired”, or ”Disabled” are asked the following question:

`occupation_past`: Which of these occupations most closely matches your main occupation?

- Student
- Agriculture / Farming
- Fishing
- Retail worker / Vendor
- Domestic labourer
- Tradesperson (e.g. construction, electrician, mechanic)
- Factory worker
- Office worker
- Security services

- Military worker
- Education worker
- Health care worker
- Communications & Information Technology worker
- Government official

`liveoutside`: Have you ever lived outside of `lowercountry` for longer than 3 months?

- Yes
- No

`deprivation`: Over the past year, how often, if ever, have you or anyone in your family gone without:

	Never	Just once or twice	Several times	Many times	Always
Enough food to eat?	o	o	o	o	o
Enough clean water for home use?	o	o	o	o	o
Medicines or medical treatment?	o	o	o	o	o
Enough fuel to cook your food?	o	o	o	o	o
Reliable electricity?	o	o	o	o	o
A cash income?	o	o	o	o	o

`ideo`: In political matters, people talk of "the left" and "the right." How would you place your views on this scale, generally speaking?

- Far left
- Left

- Center
- Right
- Far Right
- Don't know/don't have political views

Display this question if val does not equal 500:

`email`: If you would like to participate in the drawing for a chance to win US $100, please enter your email address. This is OPTIONAL.

Display this question if val equals 500:

`email500`: If you would like to participate in the drawing for a chance to win US $500, please enter your email address. This is OPTIONAL.

Display this question if val does not equal 500:

`recontact`: In the future, we may conduct a limited number of additional surveys on public opinion topics in your country. Would you like to be emailed with an invitation to these future survey opportunities? Your choice has no impact on the opportunity to win $100.

- Yes
- No

Display this question if val equals 500:

`recontact`: In the future, we may conduct a limited number of additional surveys on public opinion topics in your country. Would you like to be emailed with an invitation to these future survey opportunities? Your choice has no impact on the opportunity to win $500.

- Yes
- No

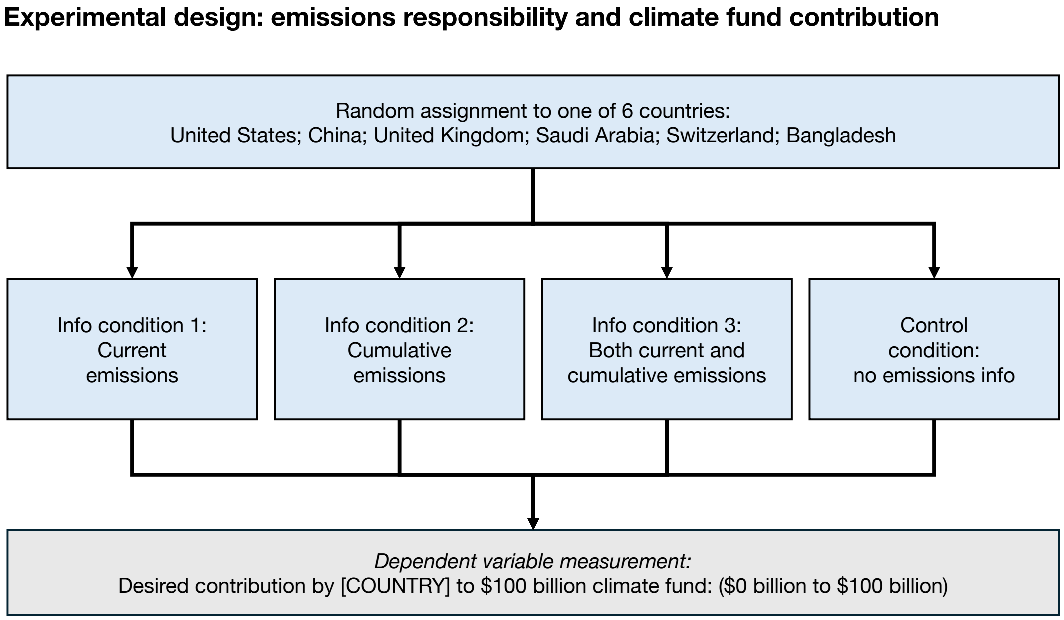

Supplementary Figure 10: **Design overview of the emissions responsibility and climate fund contribution experiment**

10 Sample sizes per quota and question block for countries and territories in sample

.

AG: Antigua and Barbuda

Quota	Target	Block 1	Block 2	Block 3	Block 4	Block 5	Finished
M 18-29	23	27	27	26	24	21	17
M 30-49	31	45	45	43	42	37	33
M 50+	30	17	17	16	16	14	12
F 18-29	24	43	42	38	35	29	23
F 30-49	37	82	82	77	70	66	56
F 50+	39	37	35	30	28	25	20
Total	184	251	248	230	215	192	161

Supplementary Table 8: **Antigua and Barbuda**. 16 respondents who came into the survey in Antigua and Barbuda self-reported living in a different country or territory (included in Table). 6 respondents self-reported living in Antigua and Barbuda but came into the survey via a different country or territory's Facebook ad (not included in Table).

AI: Anguilla (UK)

Quota	Target	Block 1	Block 2	Block 3	Block 4	Block 5	Finished
M 18-29	16	6	6	5	4	4	4
M 30-49	21	9	9	9	9	9	8
M 50+	28	7	7	7	7	7	6
F 18-29	16	6	6	6	6	6	5
F 30-49	31	12	12	12	12	12	11
F 50+	34	11	11	11	11	11	10
unknown		45	43	35	26	12	1
Total	150 [35]	96	94	85	75	61	45

Supplementary Table 9: **Anguilla**. 0.25% of eligible population is 35 respondents. The Anguillan recruitment ads had an error in their URL which prevented some metadata capture (but did not impact the survey as experienced by respondents). As a result, sample also includes 44 additional partial responses from Anguilla with unknown demographics that could not be imputed, and 1 additional complete response from a female respondent whose age could not be imputed. 6 respondents self-reported living in Anguilla but came into the survey via a different country or territory's Facebook ad (not included in Table).

AS: American Samoa (US)

Quota	Target	Block 1	Block 2	Block 3	Block 4	Block 5	Finished
M 18-29	22	22	22	20	16	13	10
M 30-49	33	41	40	34	32	29	24
M 50+	20	26	25	24	24	23	19
F 18-29	22	61	60	58	49	37	29
F 30-49	33	75	74	70	66	61	50
F 50+	20	33	32	30	30	27	22
Total	150 [81]	258	253	236	217	190	154

Supplementary Table 10: **American Samoa**. 0.25% of eligible population is 81 respondents. 3 people came into the survey via American Samoa, but self-reported living elsewhere (included in Table). 32 respondents who self-reported living in American Samoa came into the survey via a different country or territory's Facebook ad (not included in Table).

AW: Aruba (Netherlands)

Quota	Target	Block 1	Block 2	Block 3	Block 4	Block 5	Finished
M 18-29	23	13	12	10	9	9	8
M 30-49	40	37	36	34	32	29	26
M 50+	50	50	47	43	40	36	31
F 18-29	23	18	17	15	15	13	12
F 30-49	43	70	68	65	54	50	42
F 50+	64	65	63	57	51	43	37
Total	243	253	243	224	201	180	156

Supplementary Table 11: **Aruba**. 12 respondents came into the survey via Aruba, but self-reported living in a different country or territory (included in Table). 9 respondents who self-reported living in Aruba came into the survey via a different country or territory's Facebook ad (not included in Table).

BB: Barbados

Quota	Target	Block 1	Block 2	Block 3	Block 4	Block 5	Finished
capital							
M 18-29	13	13	12	10	9	6	5
M 30-49	24	21	19	18	14	13	9
M 50+	24	24	22	20	20	12	6
F 18-29	13	34	31	27	24	19	14
F 30-49	25	78	70	65	56	42	27
F 50+	29	19	17	17	14	14	11
rest							
M 18-29	28	30	29	28	26	26	22
M 30-49	55	34	32	32	31	31	28
M 50+	54	35	35	35	35	35	31
F 18-29	29	47	44	42	41	37	36
F 30-49	61	101	99	96	93	91	87
F 50+	67	51	51	50	48	48	43
Total	422	487	461	440	411	370	319

Supplementary Table 12: **Barbados**. 4 individuals who came into the survey in Barbados self-reported living elsewhere (included in Table). 37 individuals self-reported living in Barbados but came into the survey via a different country or territory's Facebook ad (not included in Table). Female respondents were easier to recruit and were oversampled closer to their initial target benchmarks, relative to male quotas which remained opened longer to hit minimums.

BM: Bermuda (UK)

Quota	Target	Block 1	Block 2	Block 3	Block 4	Block 5	Finished
M 18-29	11	11	10	8	7	7	5
M 30-49	27	27	27	24	21	21	20
M 50+	34	45	44	42	39	37	34
F 18-29	11	12	12	12	12	11	10
F 30-49	27	31	30	30	29	28	27
F 50+	40	44	44	43	41	41	38
Total	150 [131]	198	193	181	167	159	137

Supplementary Table 13: **Bermuda**. 0.25% of eligible population is 131 respondents. Some Bermudan recruitment ads had an error in their URL which prevented complete metadata capture (but did not impact the survey as experienced by respondents). As a result, sample also includes 28 additional partial responses from Bermuda with unknown demographics that could not be imputed. 7 respondents came into the survey via Bermudan ads but self-reported living elsewhere (included in Table). 3 respondents who self-reported living in Bermuda came into the survey via a different country or territory's Facebook ad (not included in Table).

BQ: Bonaire, Sint Eustatius and Saba (Netherlands)

Quota	Target	Block 1	Block 2	Block 3	Block 4	Block 5	Finished
M 18-29	13	10	9	7	7	5	4
M 30-49	29	21	19	14	13	13	9
M 50+	36	33	33	31	27	25	21
F 18-29	12	11	11	11	7	5	5
F 30-49	27	43	42	39	35	33	28
F 50+	33	31	30	26	26	24	21
Total	150 [56]	149	144	128	115	105	88

Supplementary Table 14: **Caribbean Netherlands**. 0.25% of eligible population is 56 respondents. 11 respondents who came into the survey through the Caribbean Netherlands ads self-reported living in a different place (included in Table). 3 respondents who self-reported living in the Caribbean Netherlands came into the survey via a different country or territory's Facebook ad (not included in Table).

(Additional sampling tables available upon request of authors)
